# Supplementary material for: Unlocking Kuhn Verdazyls: New Synthetic Approach and Useful Mechanistic Insights
Source: Int J Mol Sci. 2023 Jan 31;24(3):2693. doi: 10.3390/ijms24032693 (PMC9916651; doi:10.3390/ijms24032693)
Supplement: Supplementary file 1 [file ijms-24-02693-s001.zip › ijms-2167972-supplementary.pdf]

Supporting Information for

Kuhn verdazyls: new insights into the synthesis and mechanism of formation

Fedor E. Teslenko, Leonid L. Fershtat*

Laboratory of Nitrogen Compounds, N.D. Zelinsky Institute of Organic Chemistry, Russian Academy of Sciences, Leninsky prosp., 47, 119991 Moscow, Russian Federation; fershtat@ioc.ac.ru (L.L.F.)

Table of Contents

S1. EPR spectra **2**

S2. UV-Vis spectra **13**

S3. Crystallographic data **24**

S4. IR spectra **32**

S1. EPR spectra

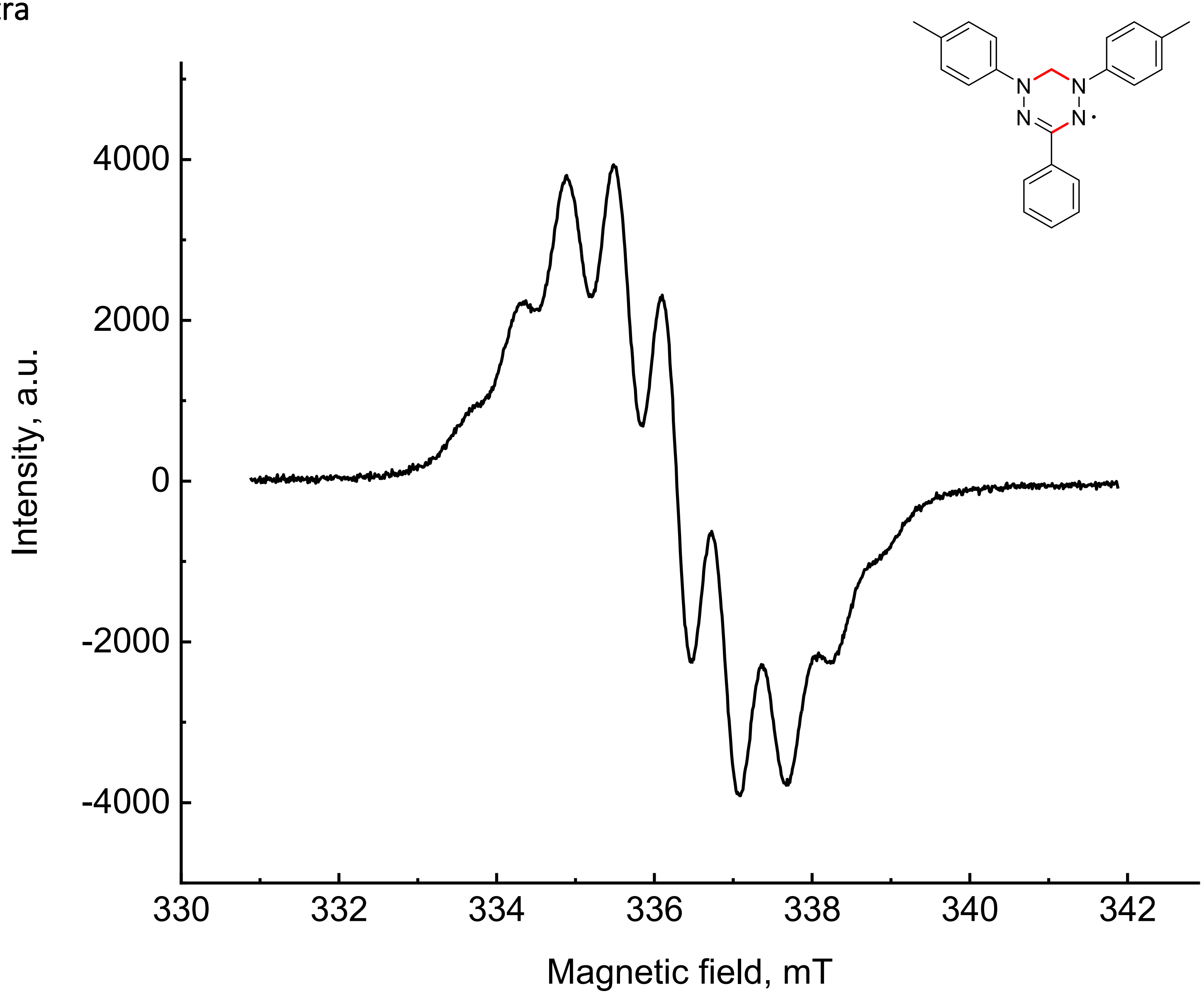

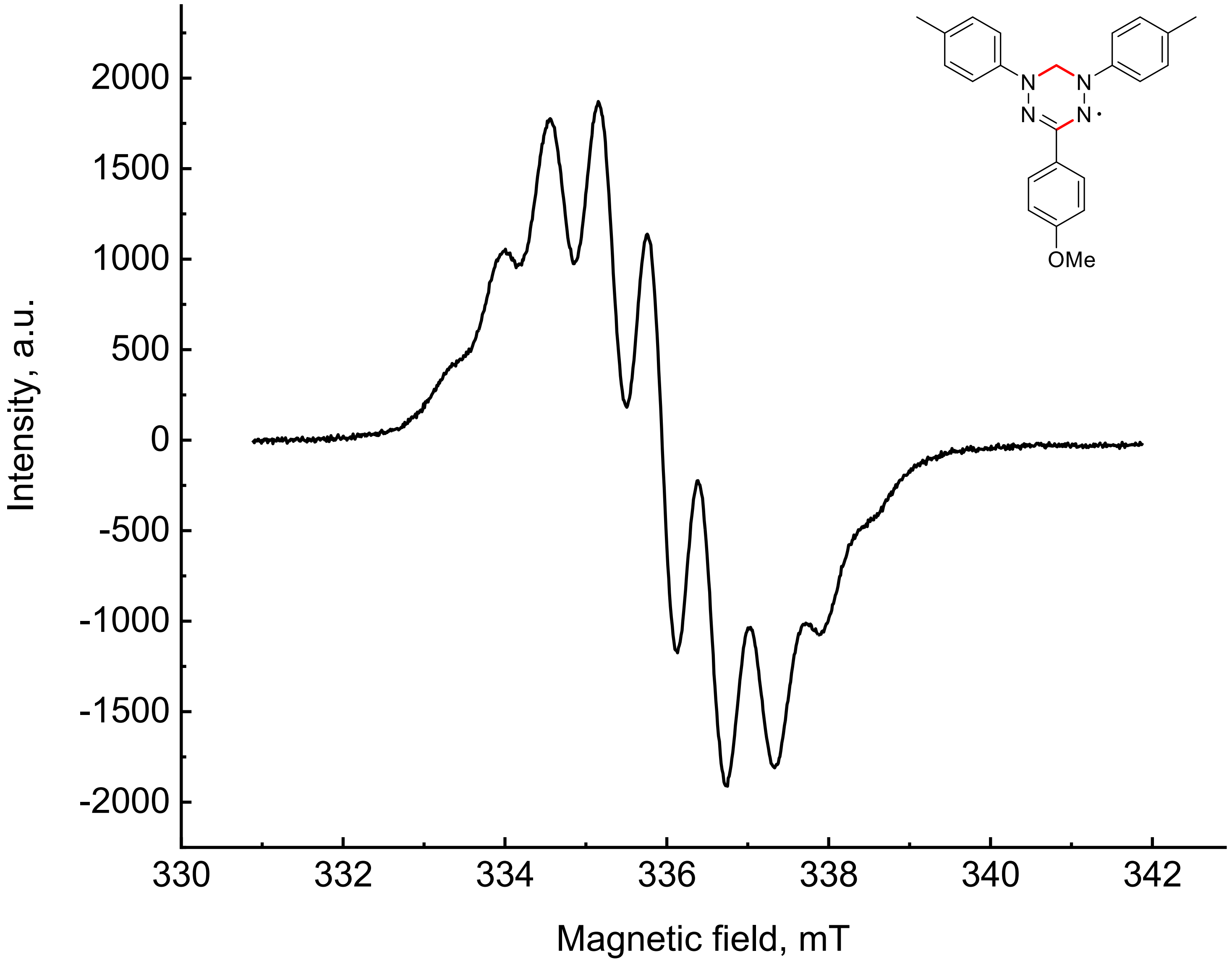

Intensity, a.u.
Magnetic field, mT
2000
1500
1000
500
0
-500
-1000
-1500
-2000
330
332
334
336
338
340
342
N
N
N
N
OMe

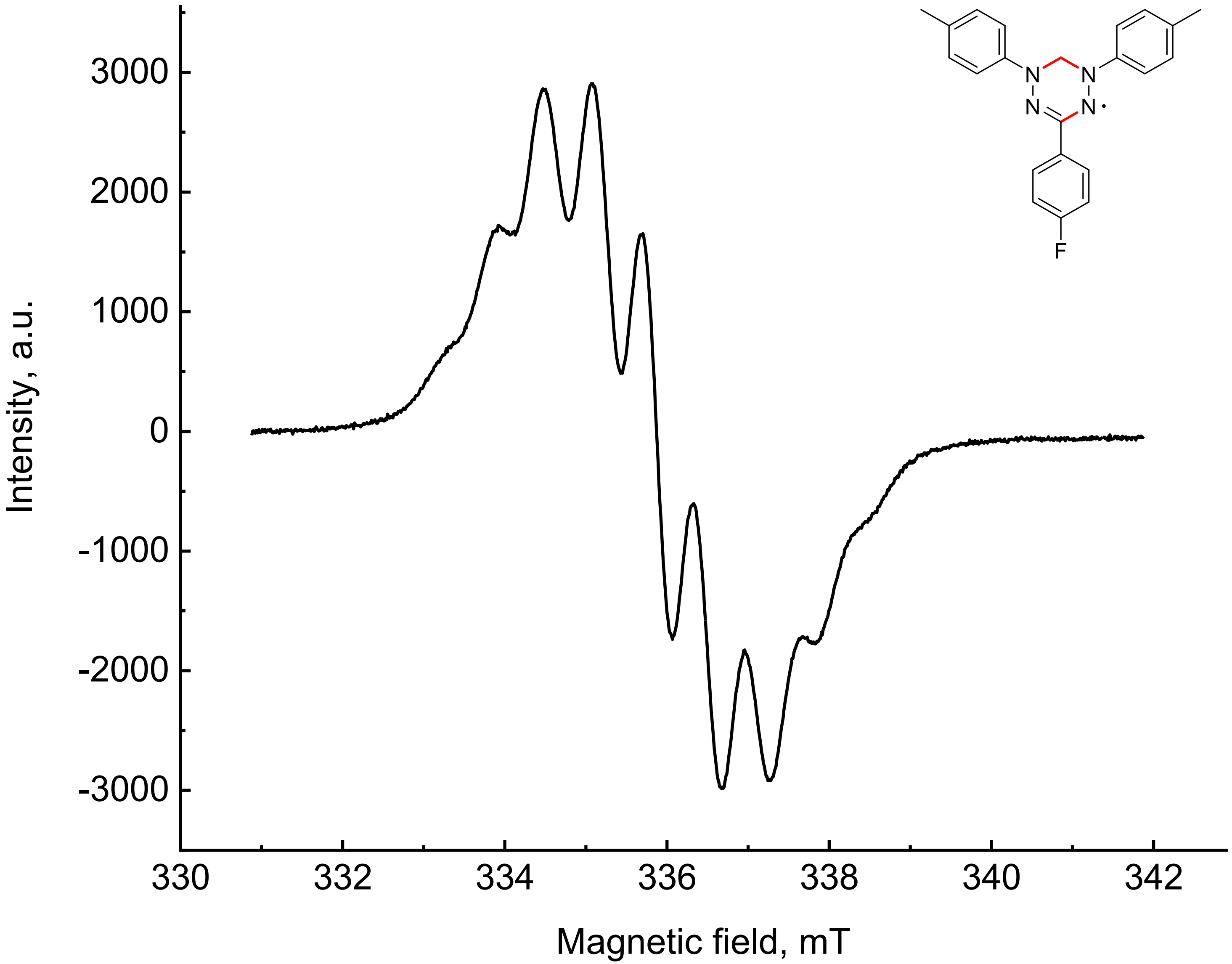

3000
2000
1000
0
-1000
-2000
-3000
Intensity, a.u.
330
332
334
336
338
340
342
Magnetic field, mT
N
N
N
N
F

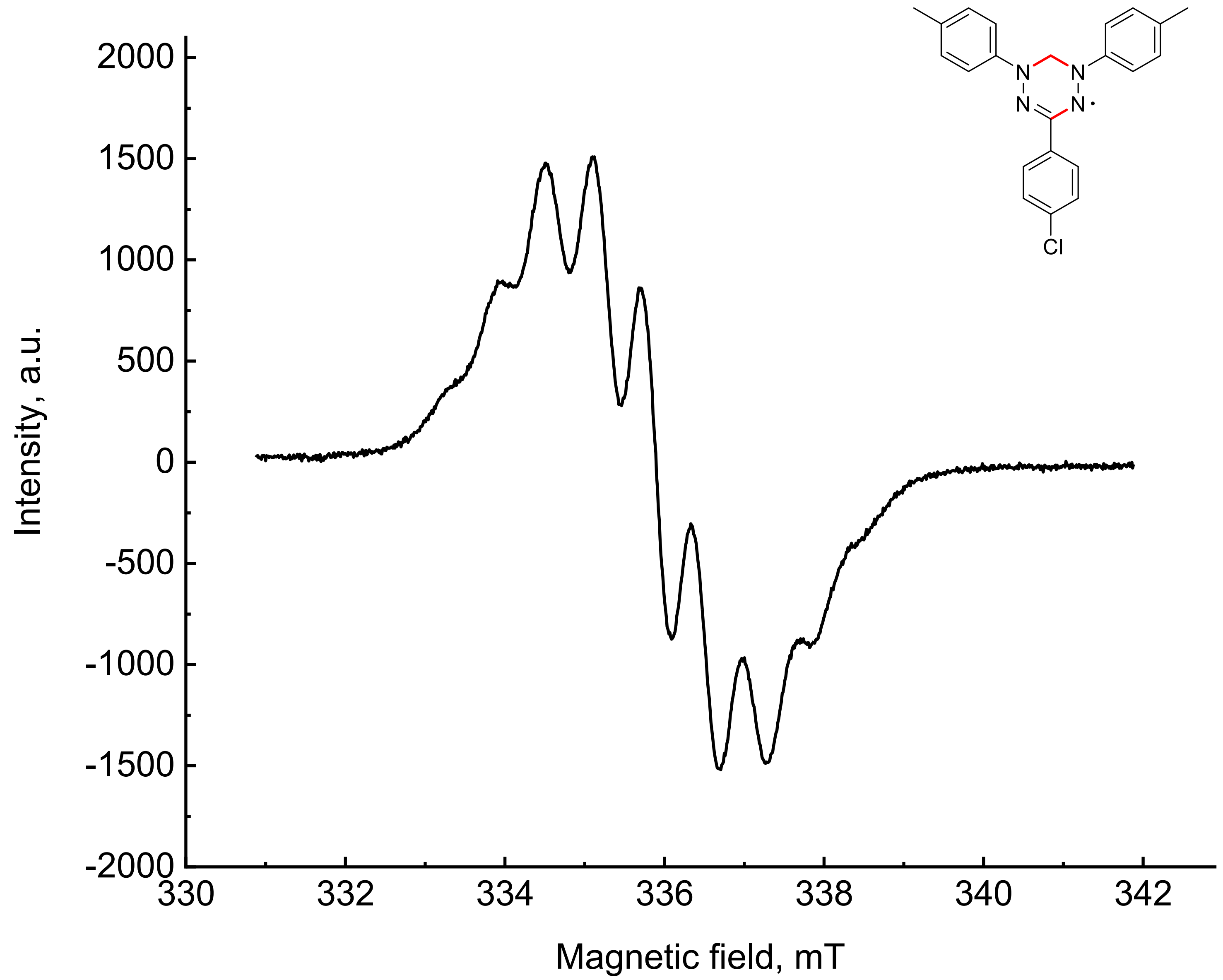

Intensity, a.u.
Magnetic field, mT
2000
1500
1000
500
0
-500
-1000
-1500
-2000
330
332
334
336
338
340
342
N
N
N
N
Cl

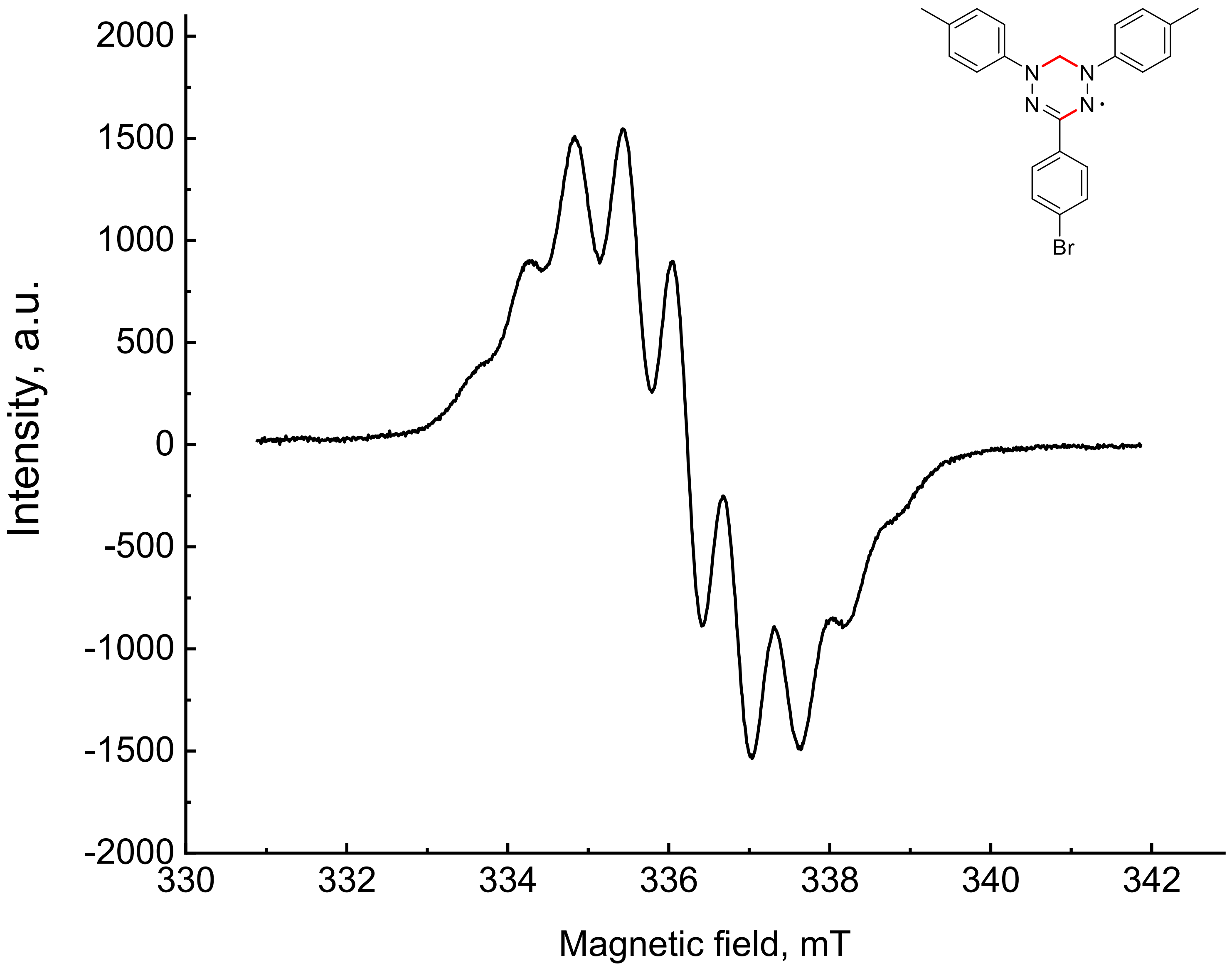

2000
1500
1000
500
0
-500
-1000
-1500
-2000
Intensity, a.u.
330
332
334
336
338
340
342
Magnetic field, mT
N
N
N
N
Br

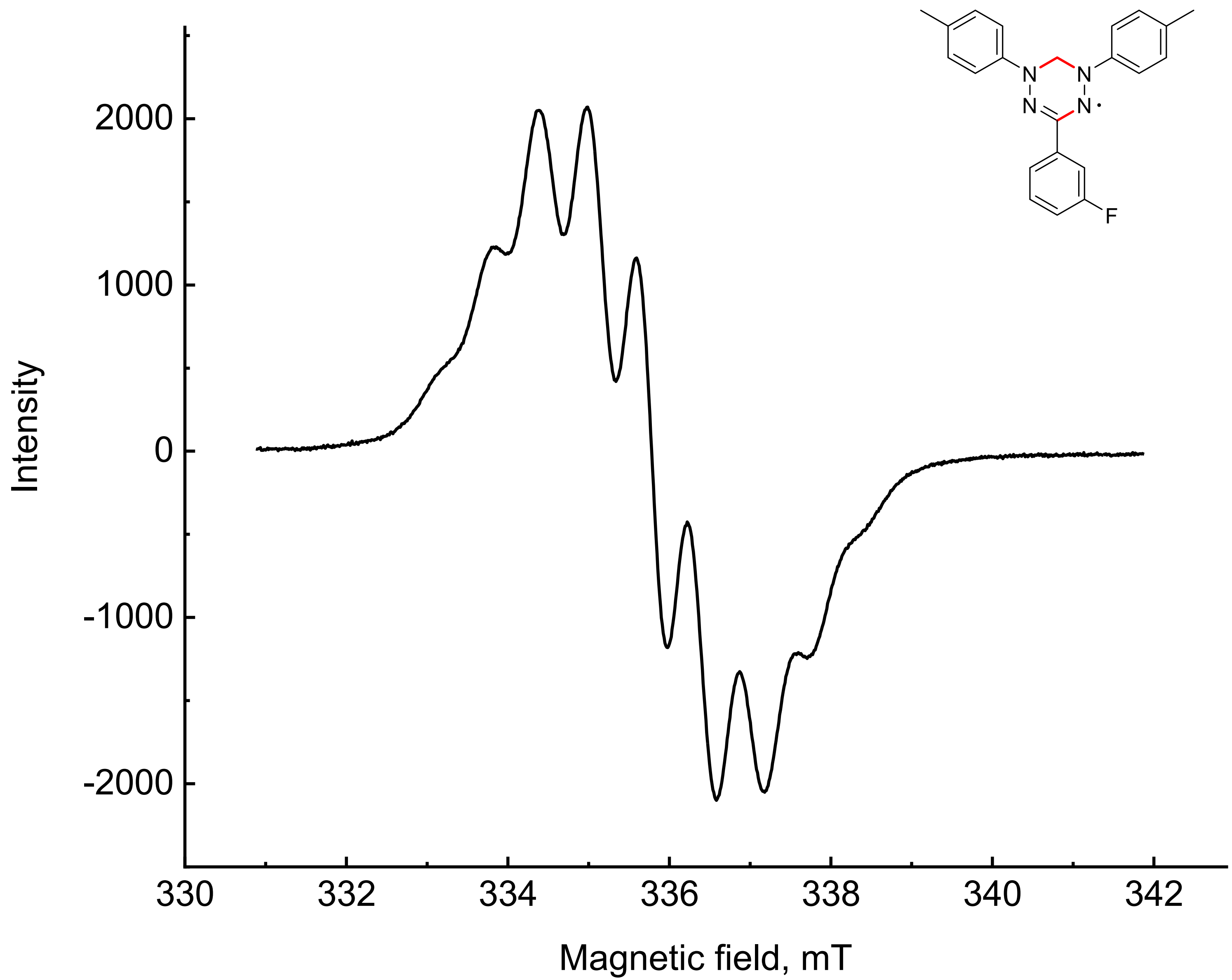

2000
1000
0
-1000
-2000
Intensity
330
332
334
336
338
340
342
Magnetic field, mT
N
N
N
N
F

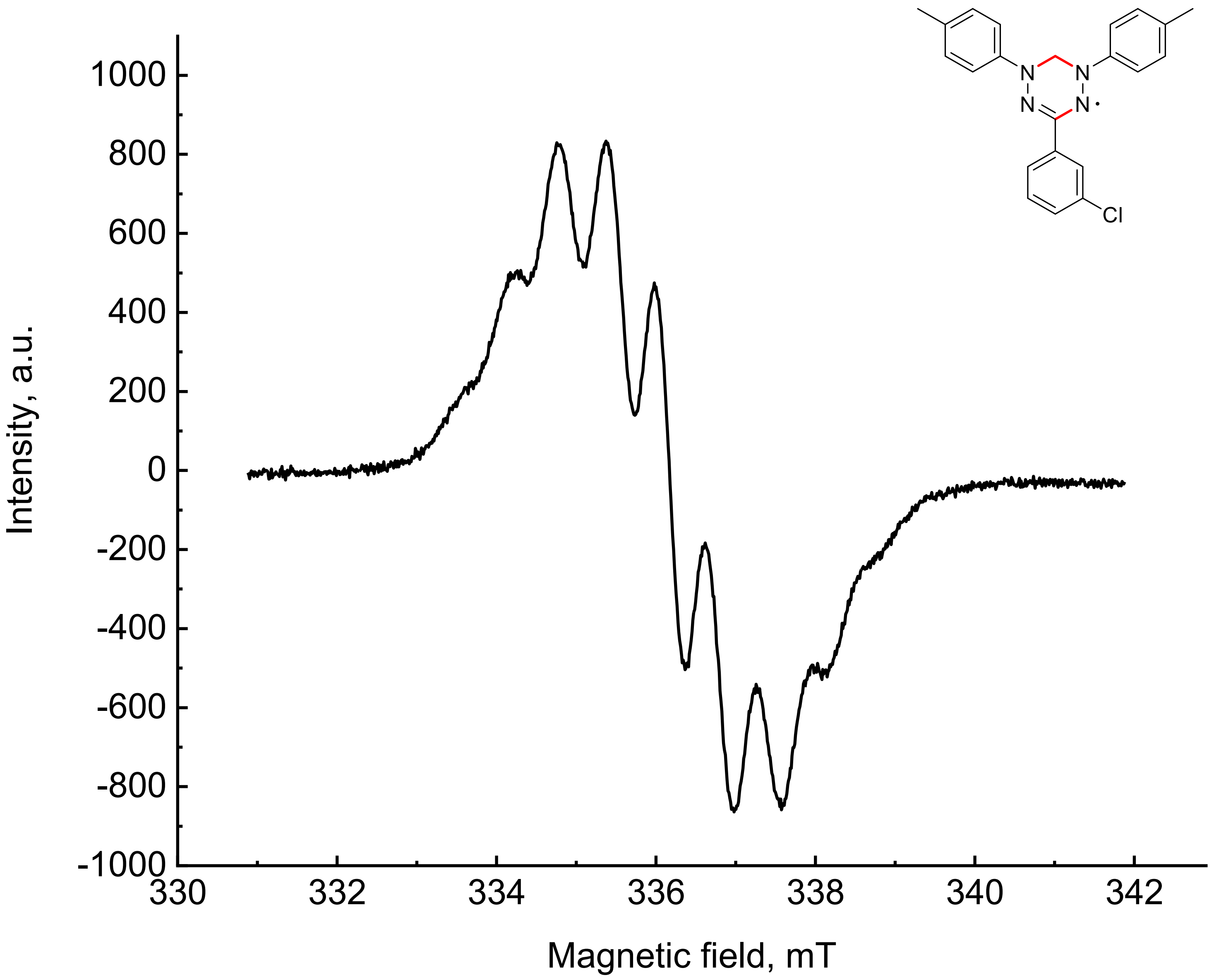

N
N
N
N·
Cl
1000
800
600
400
200
0
-200
-400
-600
-800
-1000
Intensity, a.u.
330
332
334
336
338
340
342
Magnetic field, mT

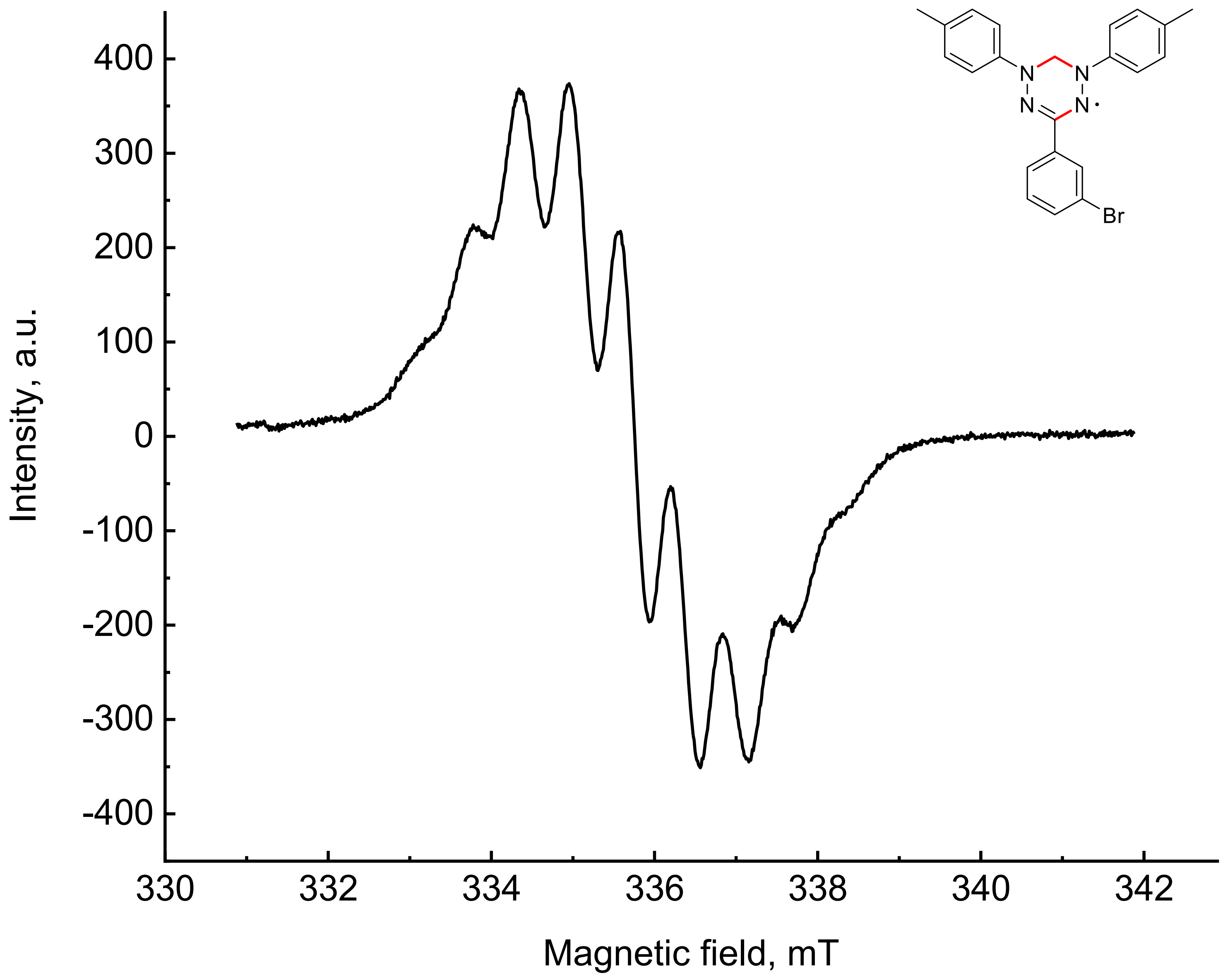
Intensity, a.u.
400
300
200
100
0
-100
-200
-300
-400
330
332
334
336
338
340
342
Magnetic field, mT
N
N
N
N
Br

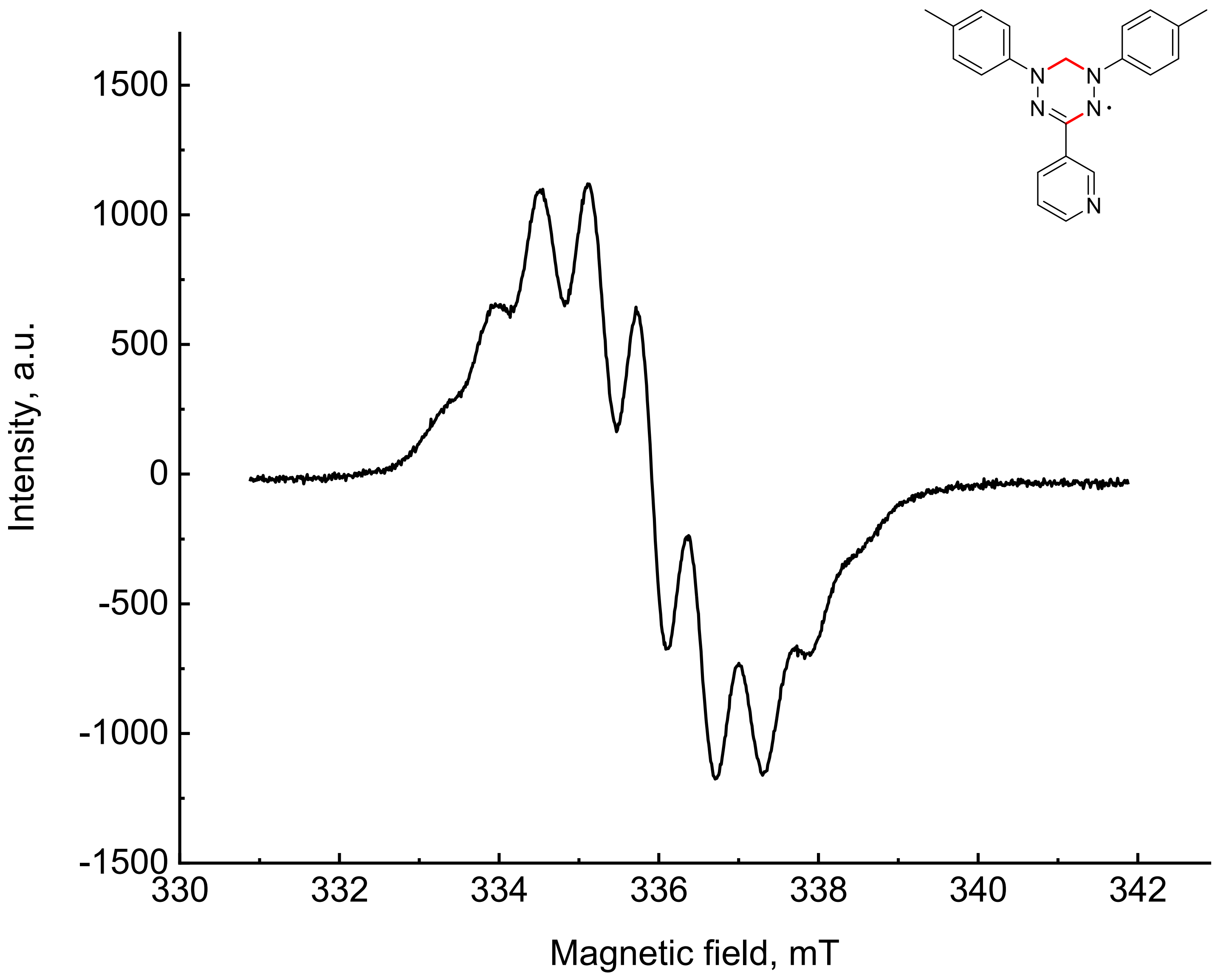
1500
1000
500
0
-500
-1000
-1500
330
332
334
336
338
340
342
Intensity, a.u.
Magnetic field, mT
N
N
N
N
N

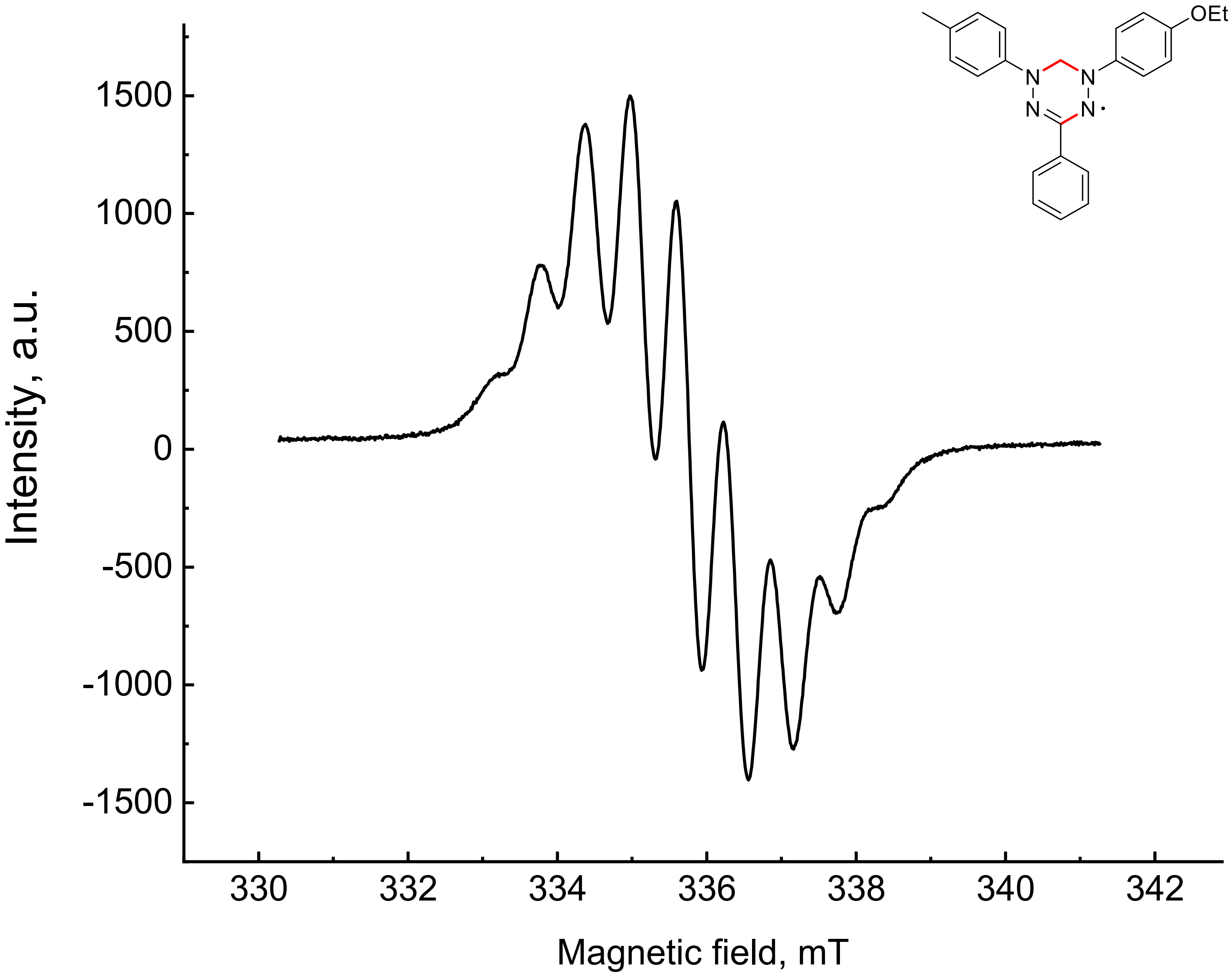

OEt
N
N
N
N·
1500
1000
500
0
-500
-1000
-1500
Intensity, a.u.
330
332
334
336
338
340
342
Magnetic field, mT

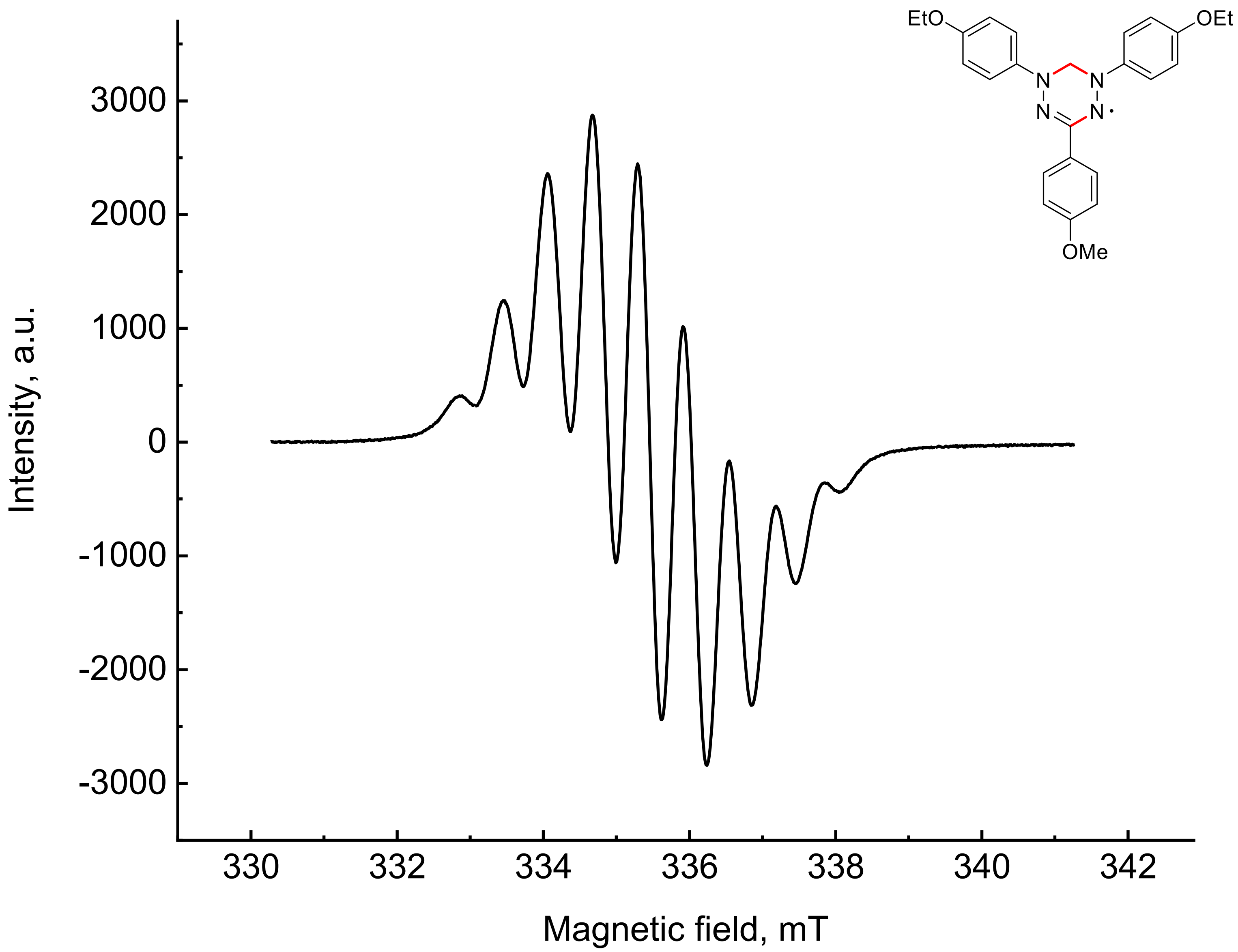

EtO
OEt
N
N
N
N
OMe
3000
2000
1000
0
-1000
-2000
-3000
Intensity, a.u.
330
332
334
336
338
340
342
Magnetic field, mT

S2. UV-Vis spectra

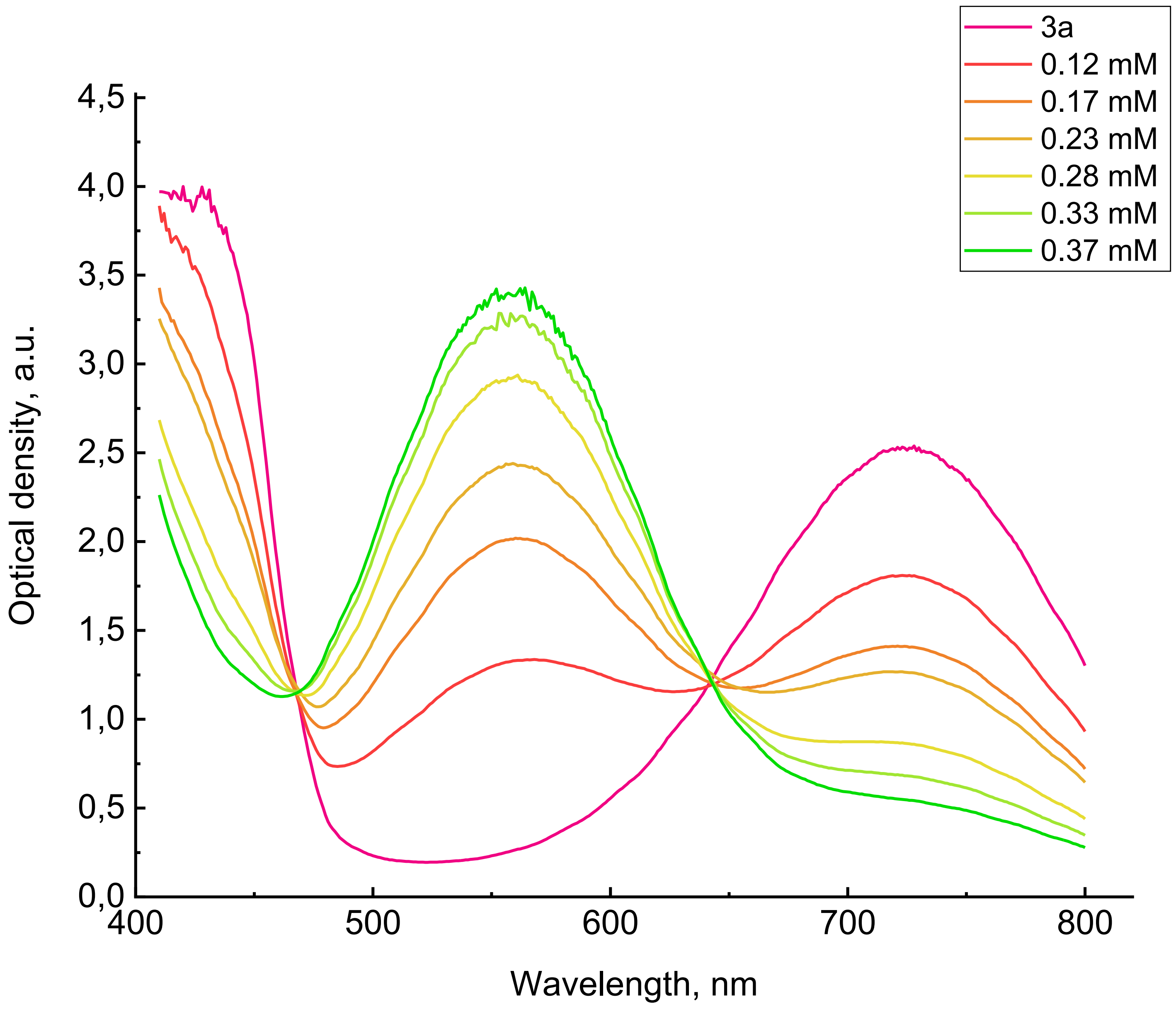

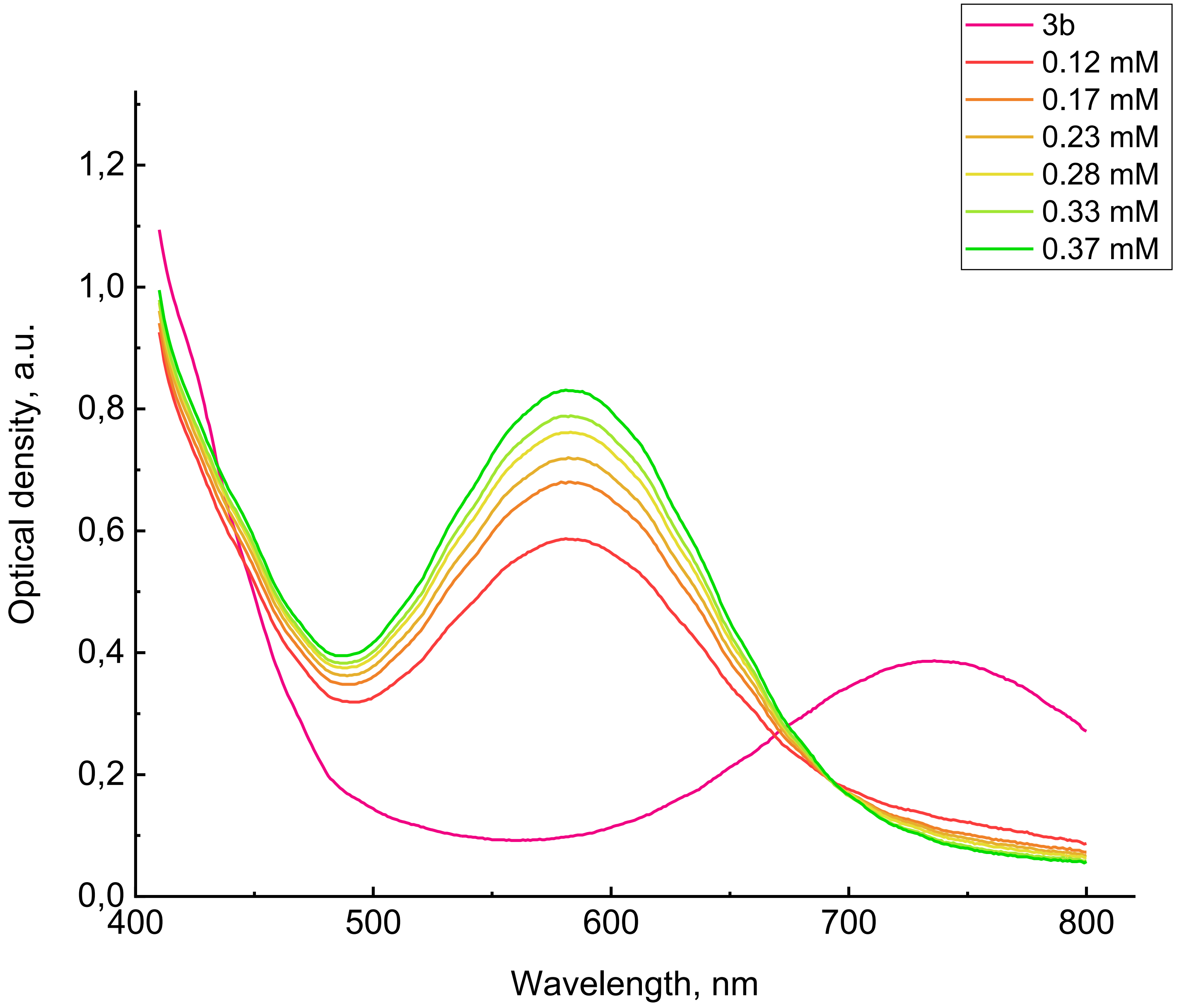
3b
0.12 mM
0.17 mM
0.23 mM
0.28 mM
0.33 mM
0.37 mM
Optical density, a.u.
0,0
0,2
0,4
0,6
0,8
1,0
1,2
400
500
600
700
800
Wavelength, nm

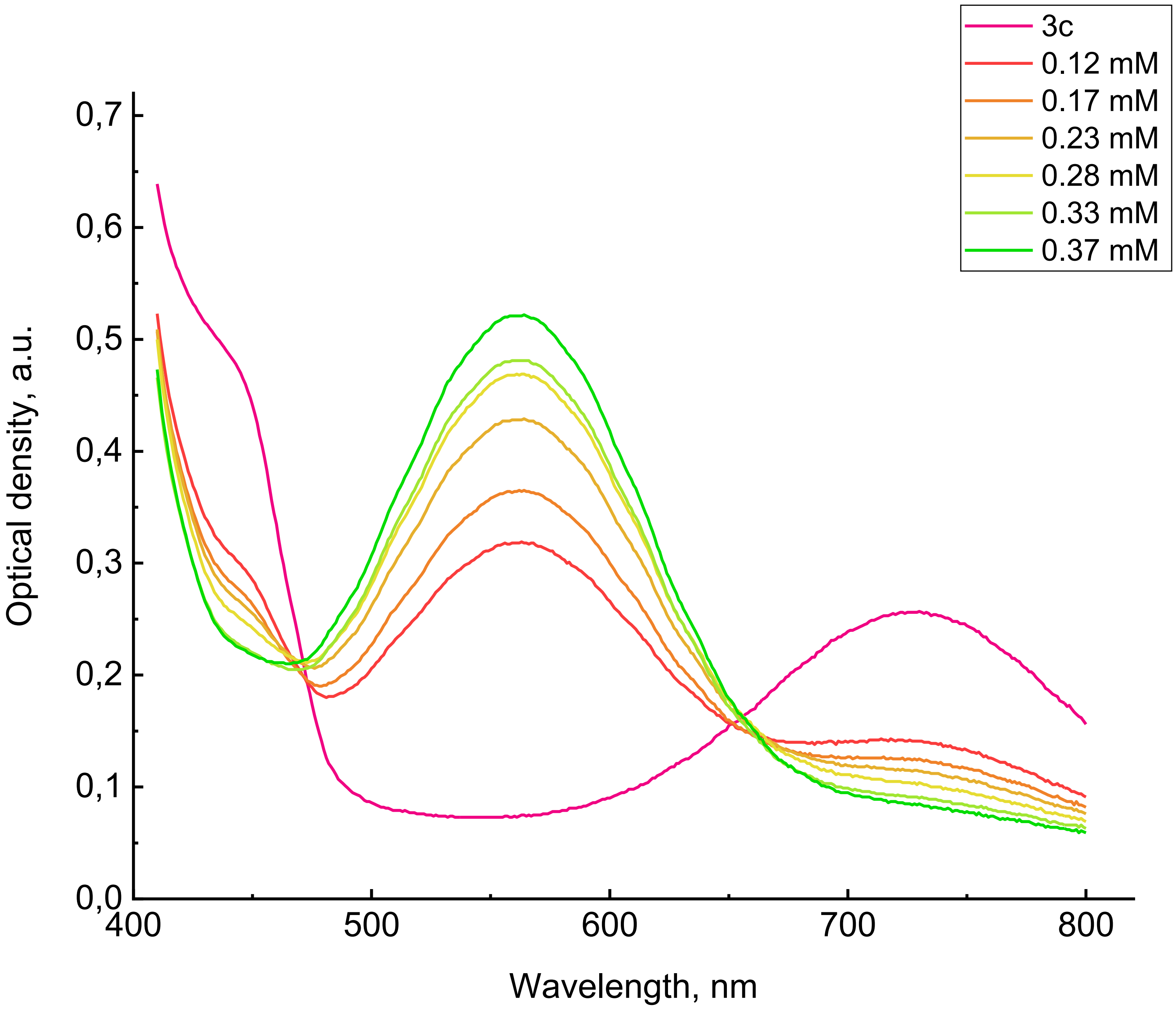
3c
0.12 mM
0.17 mM
0.23 mM
0.28 mM
0.33 mM
0.37 mM
Optical density, a.u.
0,0
0,1
0,2
0,3
0,4
0,5
0,6
0,7
400
500
600
700
800
Wavelength, nm

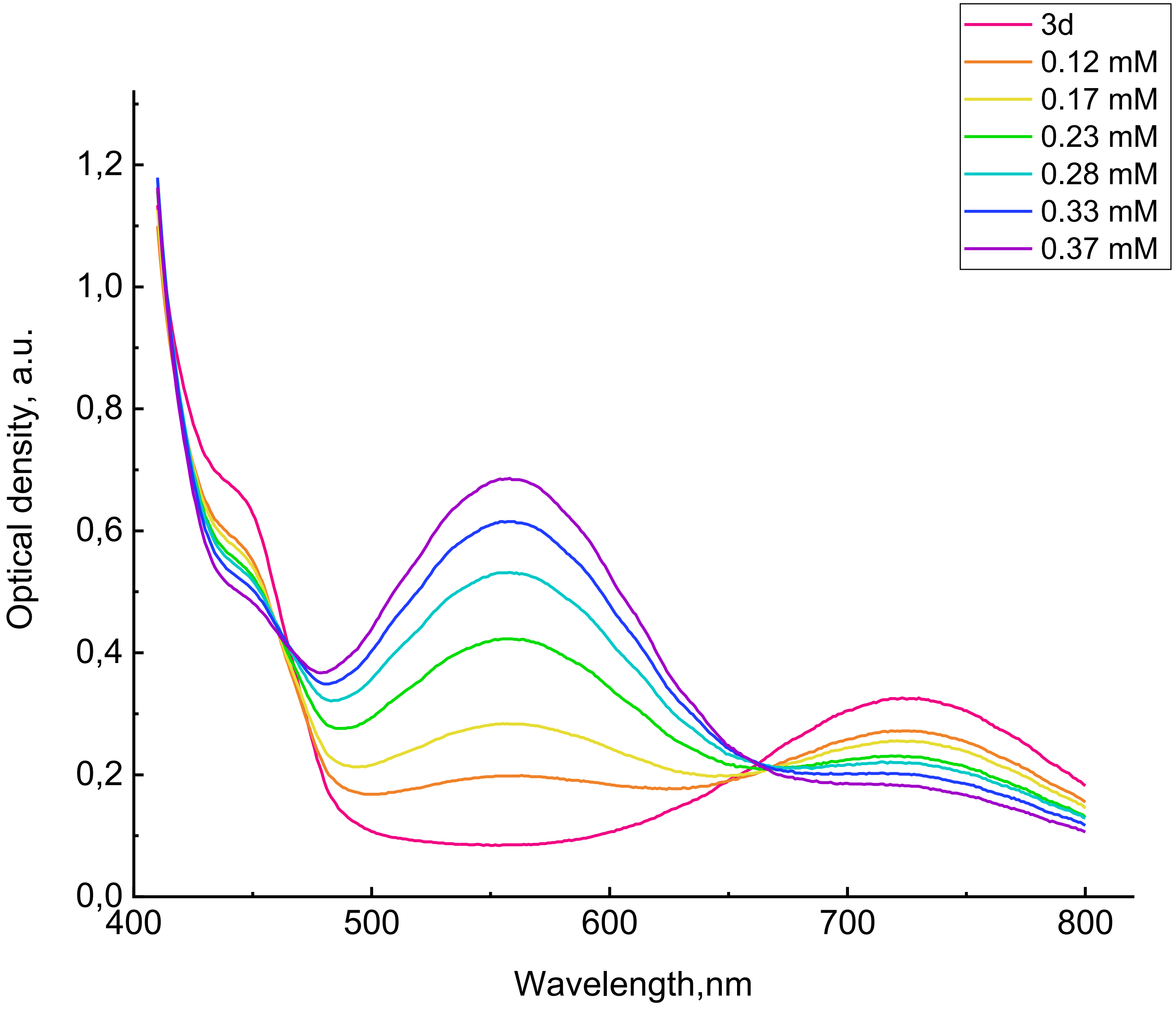

3d
0.12 mM
0.17 mM
0.23 mM
0.28 mM
0.33 mM
0.37 mM
Optical density, a.u.
0,0
0,2
0,4
0,6
0,8
1,0
1,2
400
500
600
700
800
Wavelength,nm

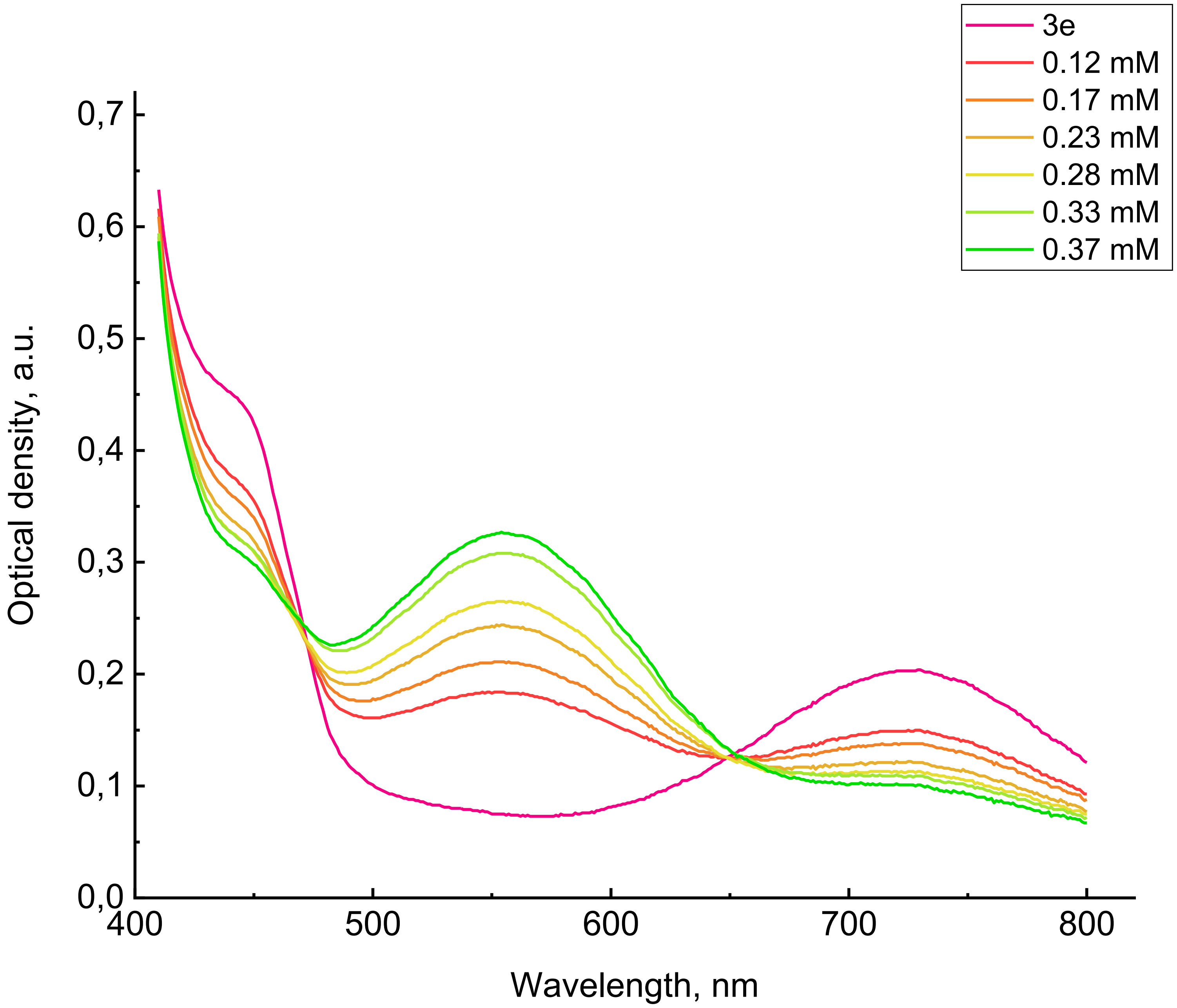

3e
0.12 mM
0.17 mM
0.23 mM
0.28 mM
0.33 mM
0.37 mM
Optical density, a.u.
0,0
0,1
0,2
0,3
0,4
0,5
0,6
0,7
400
500
600
700
800
Wavelength, nm

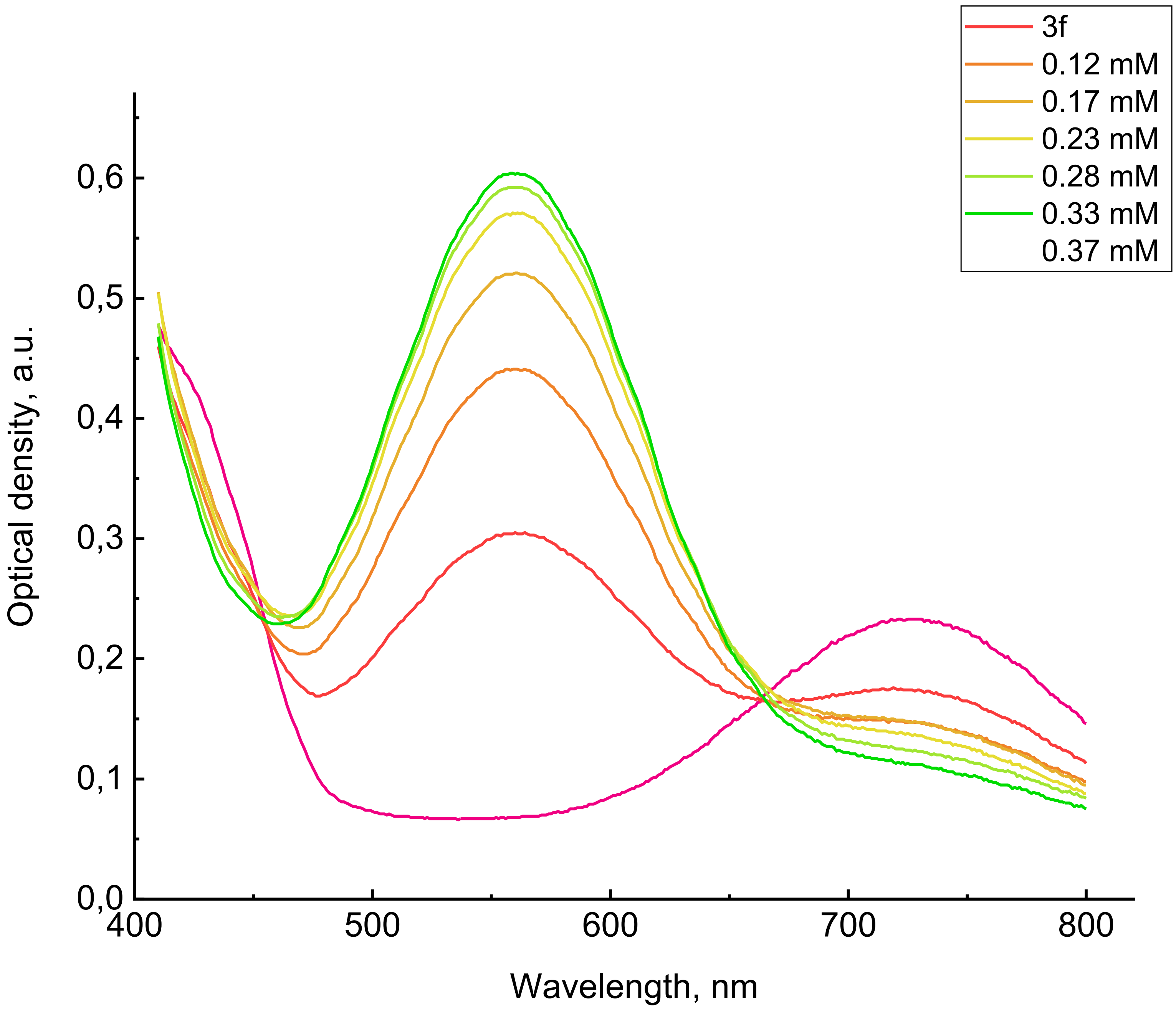

3f
0.12 mM
0.17 mM
0.23 mM
0.28 mM
0.33 mM
0.37 mM
0,0
0,1
0,2
0,3
0,4
0,5
0,6
400
500
600
700
800
Optical density, a.u.
Wavelength, nm

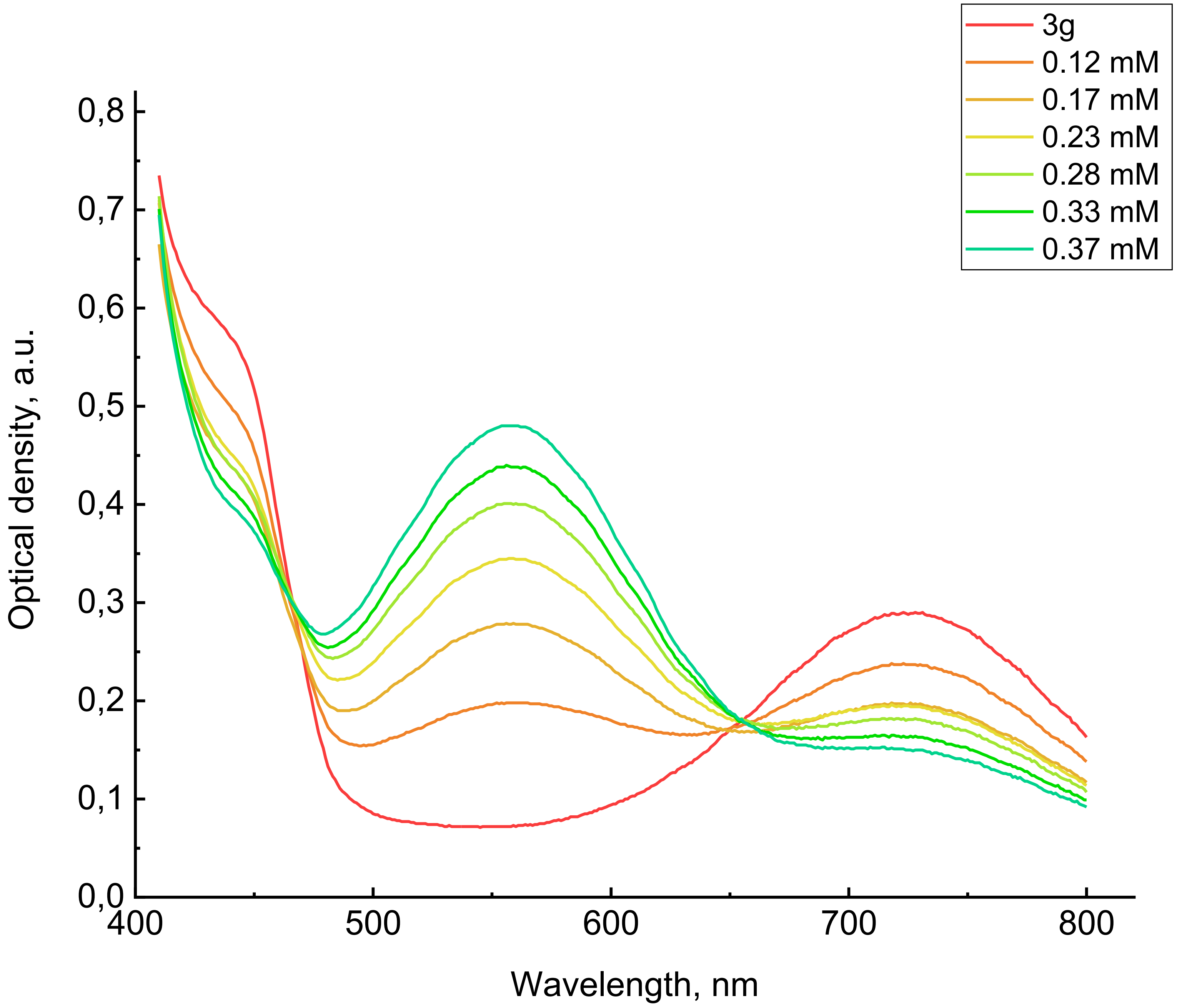
3g
0.12 mM
0.17 mM
0.23 mM
0.28 mM
0.33 mM
0.37 mM
Optical density, a.u.
0,0
0,1
0,2
0,3
0,4
0,5
0,6
0,7
0,8
400
500
600
700
800
Wavelength, nm

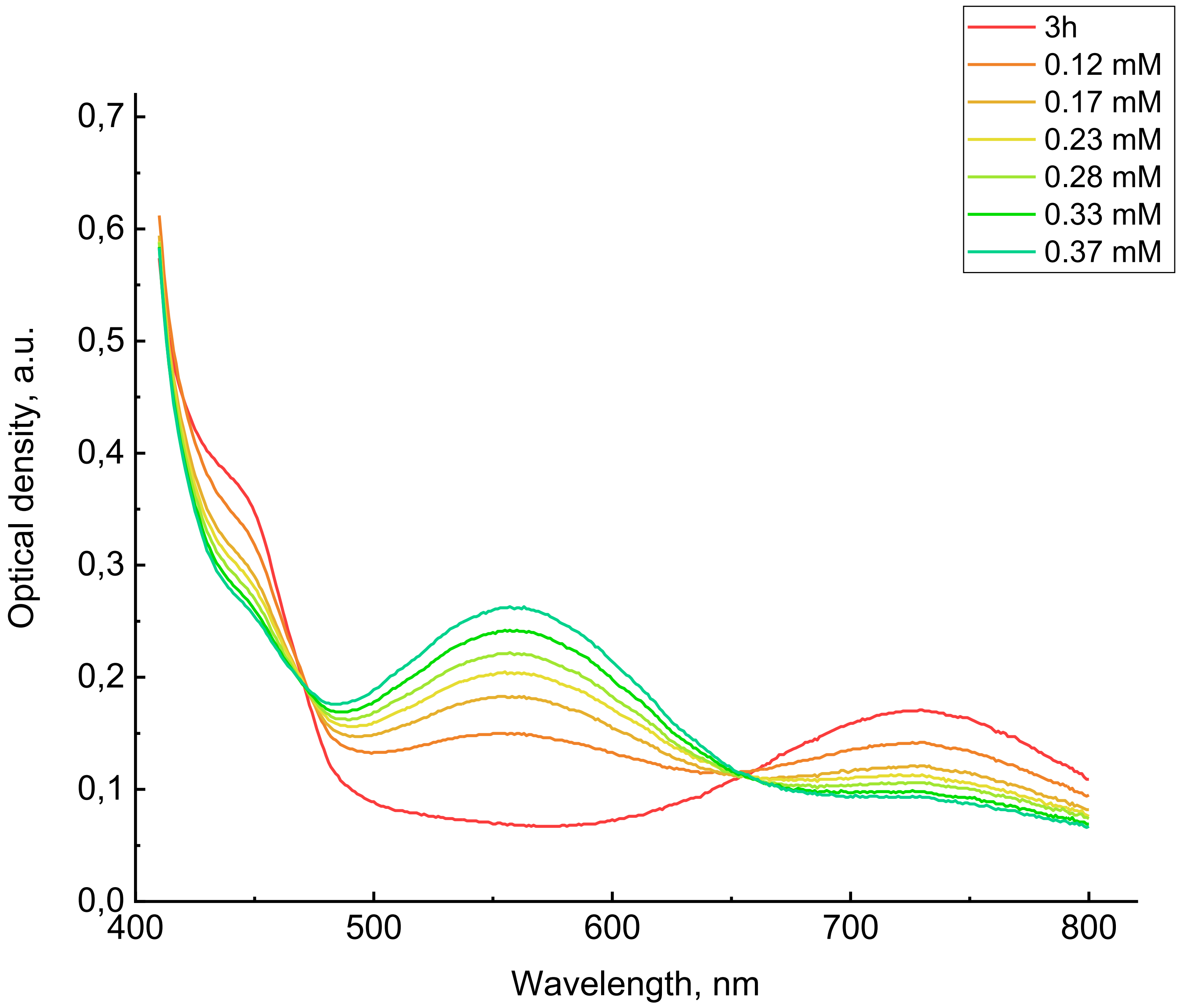

3h
0.12 mM
0.17 mM
0.23 mM
0.28 mM
0.33 mM
0.37 mM
Optical density, a.u.
0,0
0,1
0,2
0,3
0,4
0,5
0,6
0,7
400
500
600
700
800
Wavelength, nm

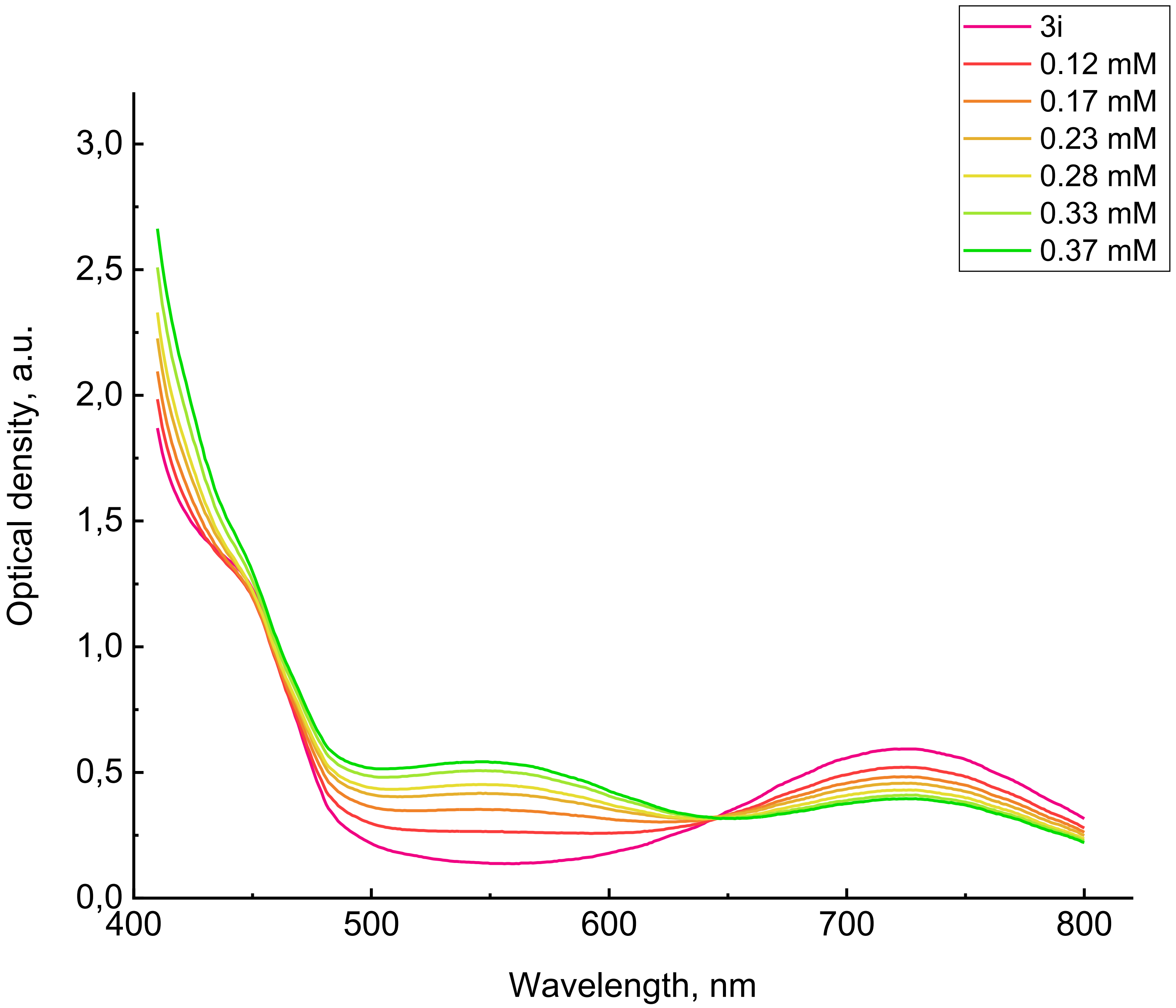
3i
0.12 mM
0.17 mM
0.23 mM
0.28 mM
0.33 mM
0.37 mM
Optical density, a.u.
3,0
2,5
2,0
1,5
1,0
0,5
0,0
400
500
600
700
800
Wavelength, nm

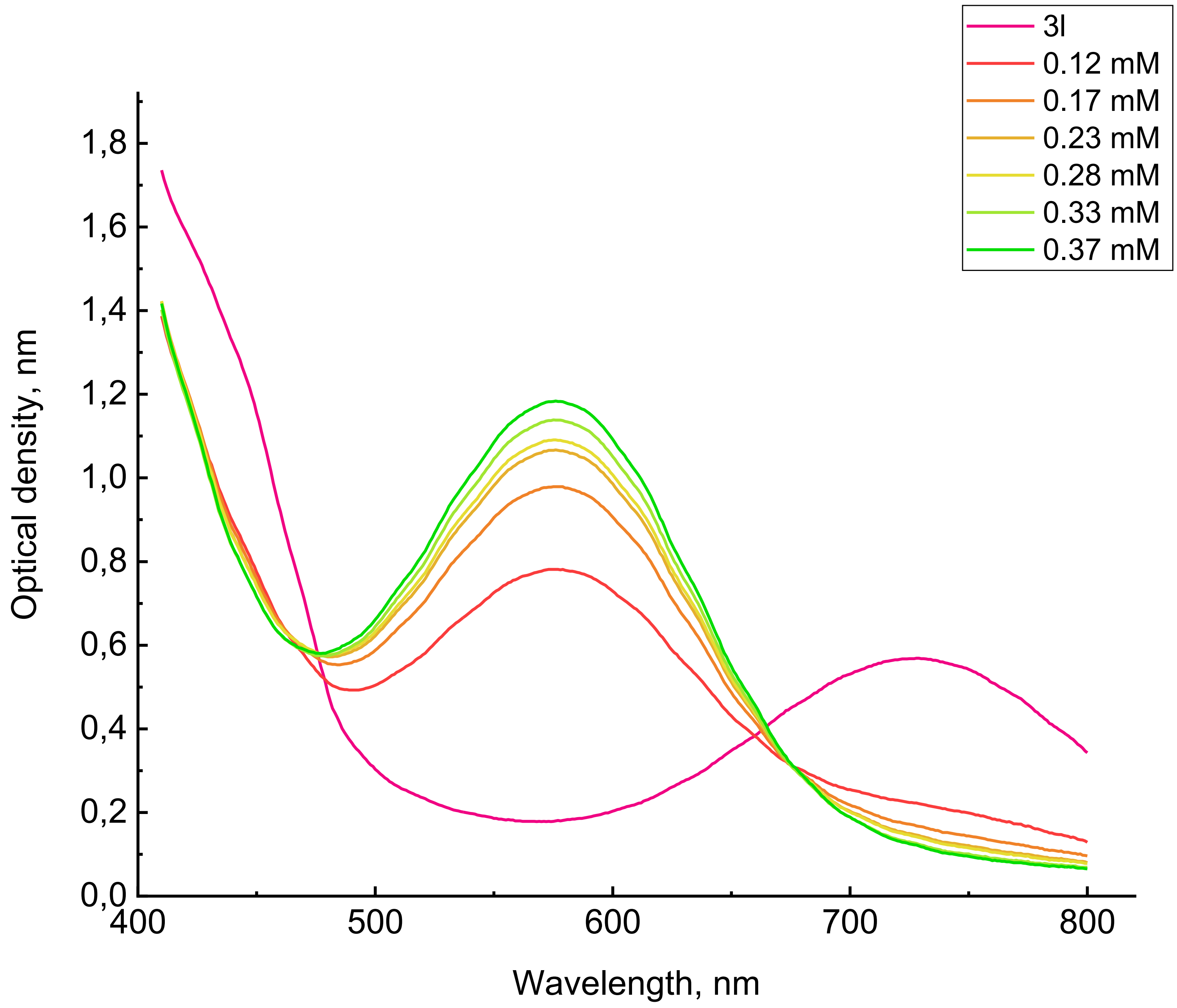
3l
0.12 mM
0.17 mM
0.23 mM
0.28 mM
0.33 mM
0.37 mM
Optical density, nm
Wavelength, nm
0,0
0,2
0,4
0,6
0,8
1,0
1,2
1,4
1,6
1,8
400
500
600
700
800

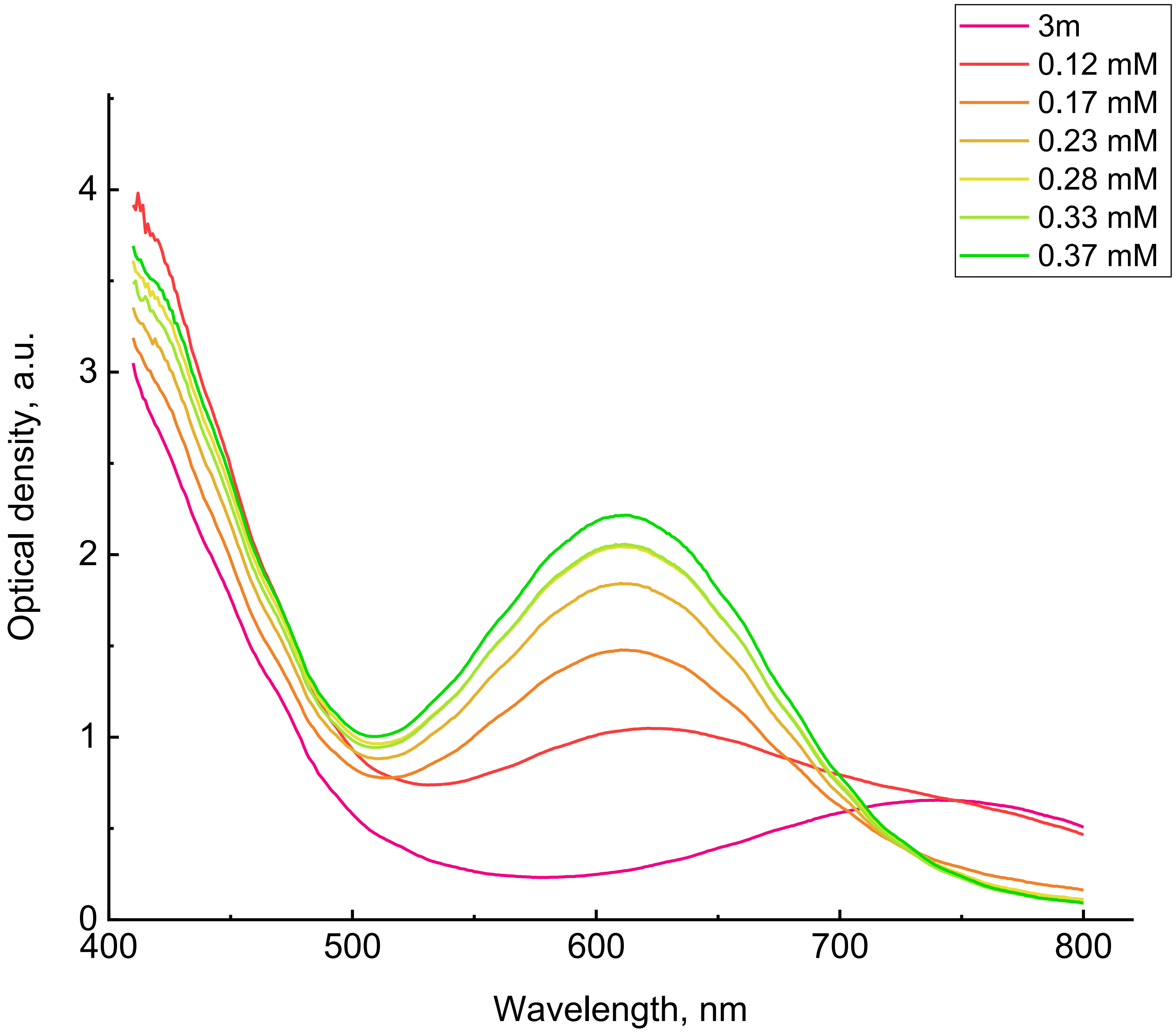

3m
0.12 mM
0.17 mM
0.23 mM
0.28 mM
0.33 mM
0.37 mM
Optical density, a.u.
0
1
2
3
4
400
500
600
700
800
Wavelength, nm

S3. Crystallographic data

Table S1. Crystal data and structure refinement for **3a**.

Empirical formula	C22 H21 N4	
Formula weight	341.43	
Temperature	100.0(1) K	
Wavelength	1.54184 Å	
Crystal system	Orthorhombic	
Space group	Pnma	
Unit cell dimensions	a = 8.71984(12) Å	α= 90°.
	b = 22.2502(4) Å	β= 90°.
	c = 9.34341(15) Å	γ = 90°.
Volume	1812.79(5) $Å^3$	
Z	4	
Density (calculated)	1.251 g/cm^3	
Absorption coefficient	0.592 mm^{-1}	
F(000)	724	
Crystal size	0.16 x 0.11 x 0.02 mm^3	
Theta range for data collection	3.974 to 79.367°.	
Index ranges	-11<=h<=11, -28<=k<=27, -11<=l<=11	
Reflections collected	24909	
Independent reflections	2009 [R(int) = 0.0441]	
Observed reflections	1878	
Completeness to theta = 67.684°	100.0 %	
Absorption correction	Semi-empirical from equivalents	
Max. and min. transmission	1.00000 and 0.60120	
Refinement method	Full-matrix least-squares on F^2	
Data / restraints / parameters	2009 / 0 / 125	

Goodness-of-fit on F^2	1.069
Final R indices [I>2sigma(I)]	R1 = 0.0518, wR2 = 0.1347
R indices (all data)	R1 = 0.0544, wR2 = 0.1367
Largest diff. peak and hole	0.300 and -0.196 e.Å^{-3}

Table S2. Atomic coordinates (x 10^4) and equivalent isotropic displacement parameters (Å^{2}x 10^3) for **3a**. U(eq) is defined as one third of the trace of the orthogonalized U^{ij} tensor.

	x	y	z	U(eq)
N(1)	4480(2)	3021(1)	5736(1)	37(1)
N(2)	5918(2)	3036(1)	5185(1)	37(1)
C(3)	6590(3)	2500	5030(2)	36(1)
C(4)	8222(3)	2500	4555(2)	36(1)
C(5)	8997(2)	3040(1)	4338(2)	42(1)
C(6)	10512(2)	3039(1)	3901(2)	49(1)
C(7)	11278(3)	2500	3678(3)	50(1)
C(8)	4047(3)	2500	6579(3)	40(1)
C(9)	3687(2)	3570(1)	5837(2)	34(1)
C(10)	2570(2)	3665(1)	6878(2)	38(1)
C(11)	1807(2)	4213(1)	6944(2)	39(1)
C(12)	2156(2)	4678(1)	6016(2)	38(1)
C(13)	3274(2)	4571(1)	4981(2)	40(1)
C(14)	4024(2)	4027(1)	4873(2)	38(1)
C(15)	1389(2)	5286(1)	6122(2)	49(1)

Table S3. Bond lengths [Å] and angles [°] for **3a**.

N(1)-N(2)	1.3557(18)
N(1)-C(8)	1.4519(18)
N(1)-C(9)	1.4055(18)
N(2)-C(3)	1.3373(16)
C(3)-N(2)#1	1.3373(16)
C(3)-C(4)	1.490(3)
C(4)-C(5)#1	1.3927(19)
C(4)-C(5)	1.3927(19)
C(5)-H(5)	0.9500
C(5)-C(6)	1.382(2)
C(6)-H(6)	0.9500
C(6)-C(7)	1.389(2)
C(7)-C(6)#1	1.389(2)
C(7)-H(7)	0.9500
C(8)-N(1)#1	1.4519(18)
C(8)-H(8A)	0.9900
C(8)-H(8B)	0.9900
C(9)-C(10)	1.393(2)
C(9)-C(14)	1.392(2)
C(10)-H(10)	0.9500
C(10)-C(11)	1.390(2)
C(11)-H(11)	0.9500
C(11)-C(12)	1.385(2)
C(12)-C(13)	1.393(2)
C(12)-C(15)	1.512(2)

C(13)-H(13)	0.9500
C(13)-C(14)	1.379(2)
C(14)-H(14)	0.9500
C(15)-H(15A)	0.9800
C(15)-H(15B)	0.9800
C(15)-H(15C)	0.9800
N(2)-N(1)-C(8)	117.80(13)
N(2)-N(1)-C(9)	117.30(12)
C(9)-N(1)-C(8)	121.92(14)
C(3)-N(2)-N(1)	115.12(13)
N(2)#1-C(3)-N(2)	126.3(2)
N(2)-C(3)-C(4)	116.80(10)
N(2)#1-C(3)-C(4)	116.80(10)
C(5)#1-C(4)-C(3)	120.46(11)
C(5)-C(4)-C(3)	120.46(11)
C(5)#1-C(4)-C(5)	119.1(2)
C(4)-C(5)-H(5)	119.8
C(6)-C(5)-C(4)	120.42(16)
C(6)-C(5)-H(5)	119.8
C(5)-C(6)-H(6)	119.8
C(5)-C(6)-C(7)	120.30(17)
C(7)-C(6)-H(6)	119.8
C(6)#1-C(7)-C(6)	119.5(2)
C(6)#1-C(7)-H(7)	120.3
C(6)-C(7)-H(7)	120.3
N(1)-C(8)-N(1)#1	105.97(18)

N(1)-C(8)-H(8A)	110.5
N(1)#1-C(8)-H(8A)	110.5
N(1)-C(8)-H(8B)	110.5
N(1)#1-C(8)-H(8B)	110.5
H(8A)-C(8)-H(8B)	108.7
C(10)-C(9)-N(1)	121.55(13)
C(14)-C(9)-N(1)	119.23(14)
C(14)-C(9)-C(10)	119.22(14)
C(9)-C(10)-H(10)	120.0
C(11)-C(10)-C(9)	119.95(14)
C(11)-C(10)-H(10)	120.0
C(10)-C(11)-H(11)	119.2
C(12)-C(11)-C(10)	121.52(15)
C(12)-C(11)-H(11)	119.2
C(11)-C(12)-C(13)	117.43(14)
C(11)-C(12)-C(15)	122.02(15)
C(13)-C(12)-C(15)	120.53(14)
C(12)-C(13)-H(13)	118.9
C(14)-C(13)-C(12)	122.19(14)
C(14)-C(13)-H(13)	118.9
C(9)-C(14)-H(14)	120.2
C(13)-C(14)-C(9)	119.66(15)
C(13)-C(14)-H(14)	120.2
C(12)-C(15)-H(15A)	109.5
C(12)-C(15)-H(15B)	109.5
C(12)-C(15)-H(15C)	109.5
H(15A)-C(15)-H(15B)	109.5

H(15A)-C(15)-H(15C)	109.5
H(15B)-C(15)-H(15C)	109.5

Symmetry transformations used to generate equivalent atoms:

#1 x,-y+1/2,z

Table S4. Anisotropic displacement parameters ($Å^2$x 10^3) for **3a**. The anisotropic displacement factor exponent takes the form: $-2\pi^2[h^2 a^{*2}U^{11} + ... + 2 h k a^* b^* U^{12}]$

	U^{11}	U^{22}	U^{33}	U^{23}	U^{13}	U^{12}
N(1)	39(1)	26(1)	46(1)	1(1)	2(1)	0(1)
N(2)	37(1)	33(1)	40(1)	-2(1)	0(1)	-1(1)
C(3)	42(1)	32(1)	36(1)	0	-4(1)	0
C(4)	42(1)	32(1)	34(1)	0	-5(1)	0
C(5)	42(1)	36(1)	47(1)	3(1)	-2(1)	-2(1)
C(6)	44(1)	52(1)	49(1)	7(1)	0(1)	-8(1)
C(7)	37(1)	74(2)	40(1)	0	2(1)	0
C(8)	46(1)	27(1)	46(1)	0	5(1)	0
C(9)	36(1)	25(1)	42(1)	-2(1)	-4(1)	0(1)
C(10)	49(1)	26(1)	40(1)	0(1)	3(1)	-4(1)
C(11)	44(1)	30(1)	44(1)	-6(1)	5(1)	-2(1)
C(12)	41(1)	27(1)	44(1)	-2(1)	-4(1)	1(1)
C(13)	44(1)	30(1)	45(1)	6(1)	0(1)	-1(1)
C(14)	36(1)	33(1)	44(1)	2(1)	2(1)	0(1)
C(15)	61(1)	30(1)	55(1)	-3(1)	-4(1)	6(1)

Table S5. Hydrogen coordinates (x 10^4) and isotropic displacement parameters ($Å^2$x 10^3) for **3a**.

	x	y	z	U(eq)
H(5)	8482	3410	4491	50
H(6)	11031	3409	3753	58
H(7)	12318	2500	3375	60
H(8A)	2929	2500	6764	47
H(8B)	4593	2500	7508	47
H(10)	2330	3356	7543	46
H(11)	1028	4269	7643	47
H(13)	3527	4883	4328	48
H(14)	4766	3966	4143	46
H(15A)	2036	5559	6683	73
H(15B)	391	5242	6593	73
H(15C)	1242	5452	5160	73

Table S6. Torsion angles [°] for **3a**.

N(1)-N(2)-C(3)-N(2)#1	9.3(3)
N(1)-N(2)-C(3)-C(4)	-174.56(15)
N(1)-C(9)-C(10)-C(11)	-179.85(14)
N(1)-C(9)-C(14)-C(13)	-178.76(14)
N(2)-N(1)-C(8)-N(1)#1	-49.3(2)
N(2)-N(1)-C(9)-C(10)	-151.90(14)
N(2)-N(1)-C(9)-C(14)	28.3(2)
N(2)-C(3)-C(4)-C(5)#1	-178.64(18)
N(2)#1-C(3)-C(4)-C(5)	178.64(18)
N(2)#1-C(3)-C(4)-C(5)#1	-2.1(3)
N(2)-C(3)-C(4)-C(5)	2.1(3)
C(3)-C(4)-C(5)-C(6)	179.83(18)
C(4)-C(5)-C(6)-C(7)	-0.2(3)
C(5)#1-C(4)-C(5)-C(6)	0.6(3)
C(5)-C(6)-C(7)-C(6)#1	-0.2(4)
C(8)-N(1)-N(2)-C(3)	23.0(2)
C(8)-N(1)-C(9)-C(10)	8.1(2)
C(8)-N(1)-C(9)-C(14)	-171.70(16)
C(9)-N(1)-N(2)-C(3)	-176.17(15)
C(9)-N(1)-C(8)-N(1)#1	150.80(11)
C(9)-C(10)-C(11)-C(12)	-1.6(2)
C(10)-C(9)-C(14)-C(13)	1.4(2)
C(10)-C(11)-C(12)-C(13)	1.8(2)
C(10)-C(11)-C(12)-C(15)	-177.00(15)
C(11)-C(12)-C(13)-C(14)	-0.4(2)

C(12)-C(13)-C(14)-C(9)	-1.2(2)
C(14)-C(9)-C(10)-C(11)	0.0(2)
C(15)-C(12)-C(13)-C(14)	178.45(15)

Symmetry transformations used to generate equivalent atoms:

#1 x,-y+1/2,z

S4. IR spectra

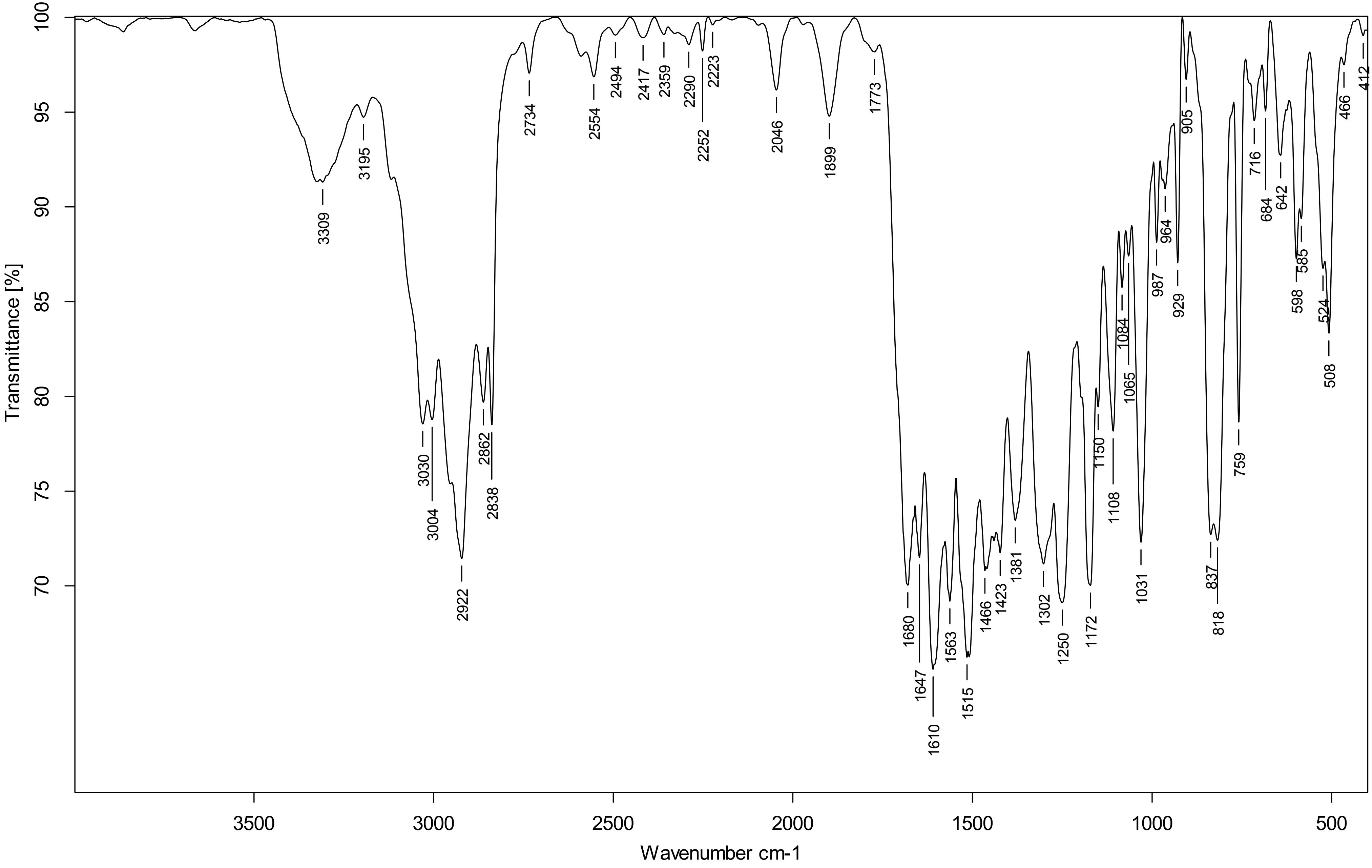

3b KBr | 18.02.2022

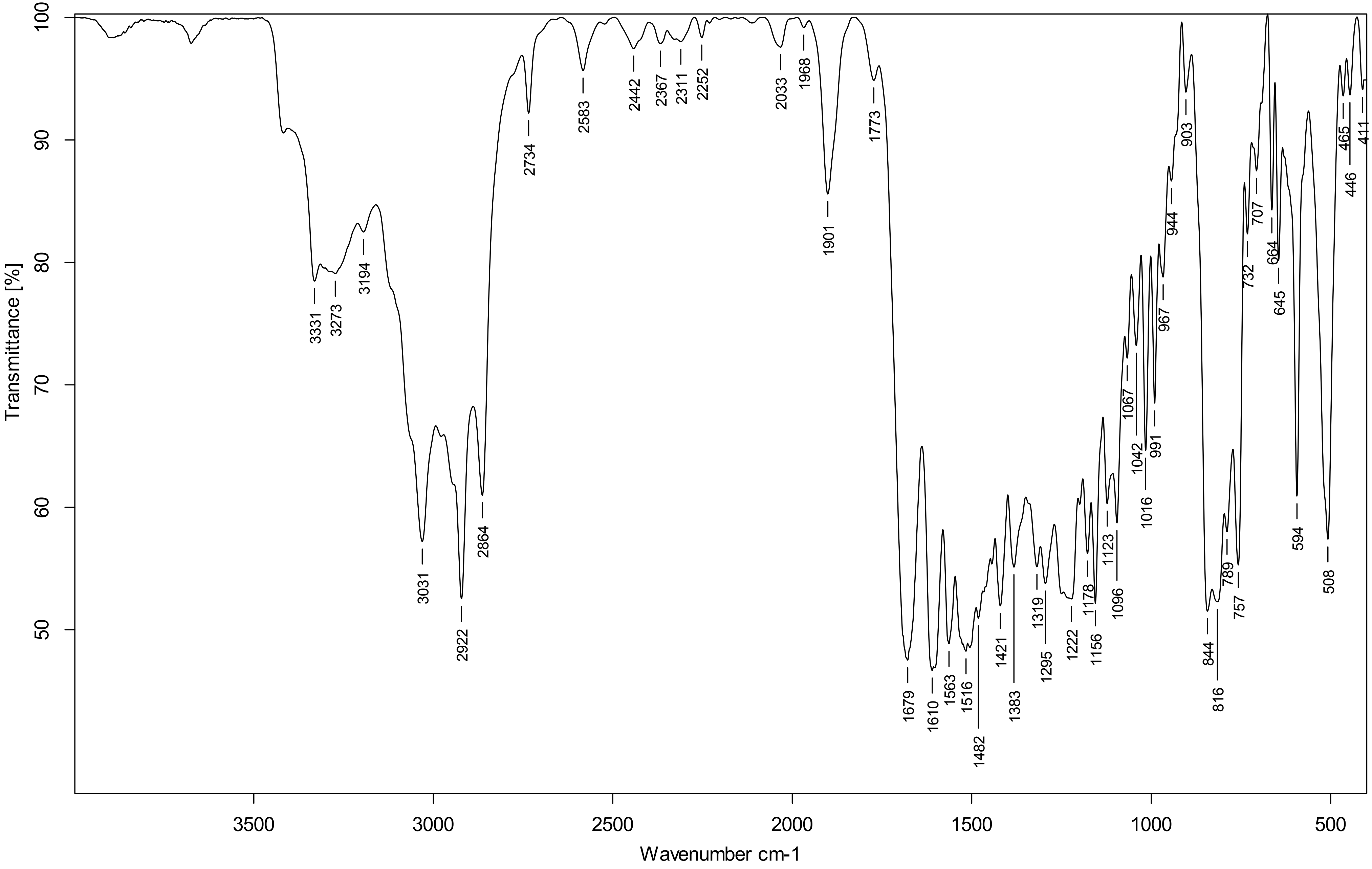

**3c** KBr	18.02.2022

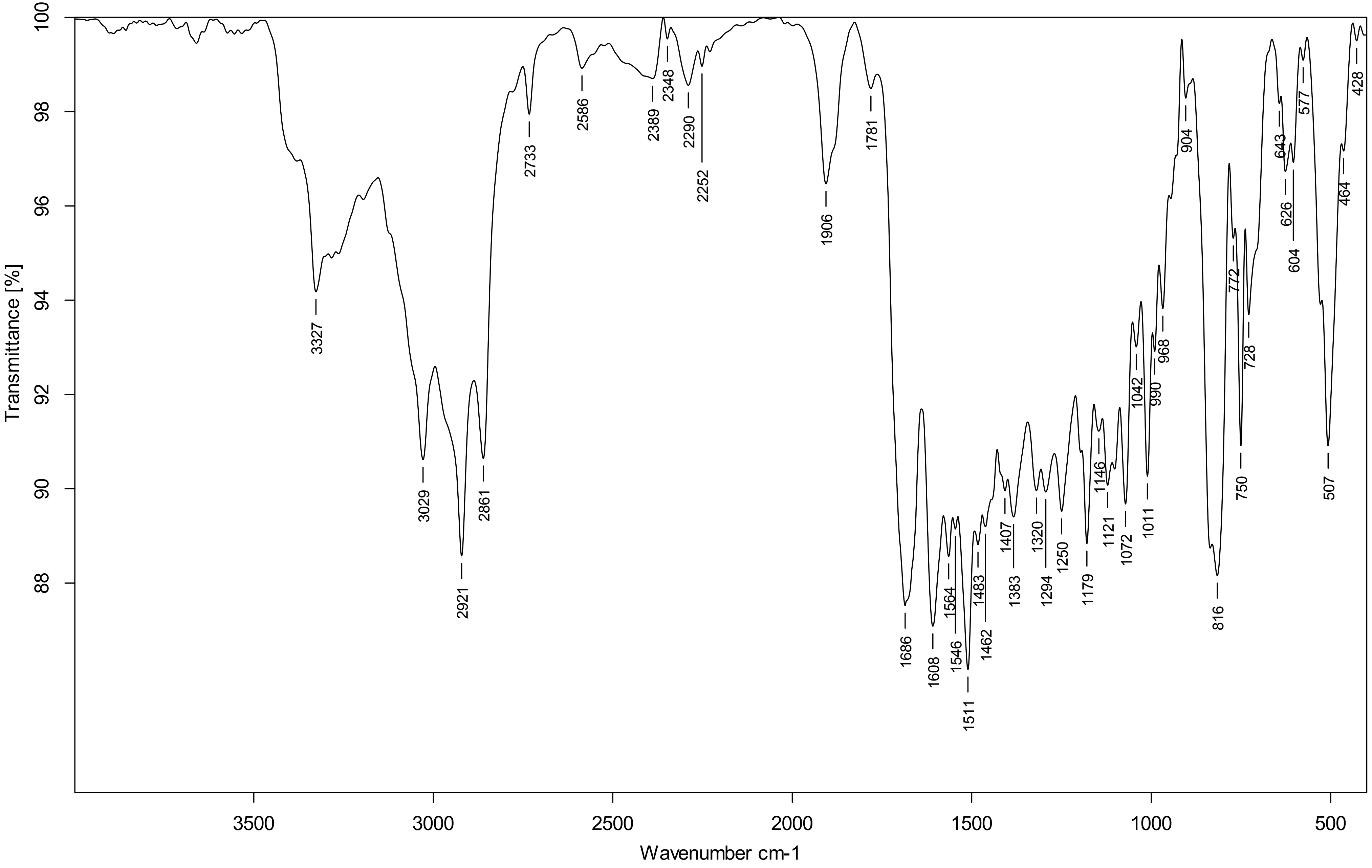

**3e** KBr	18.02.2022

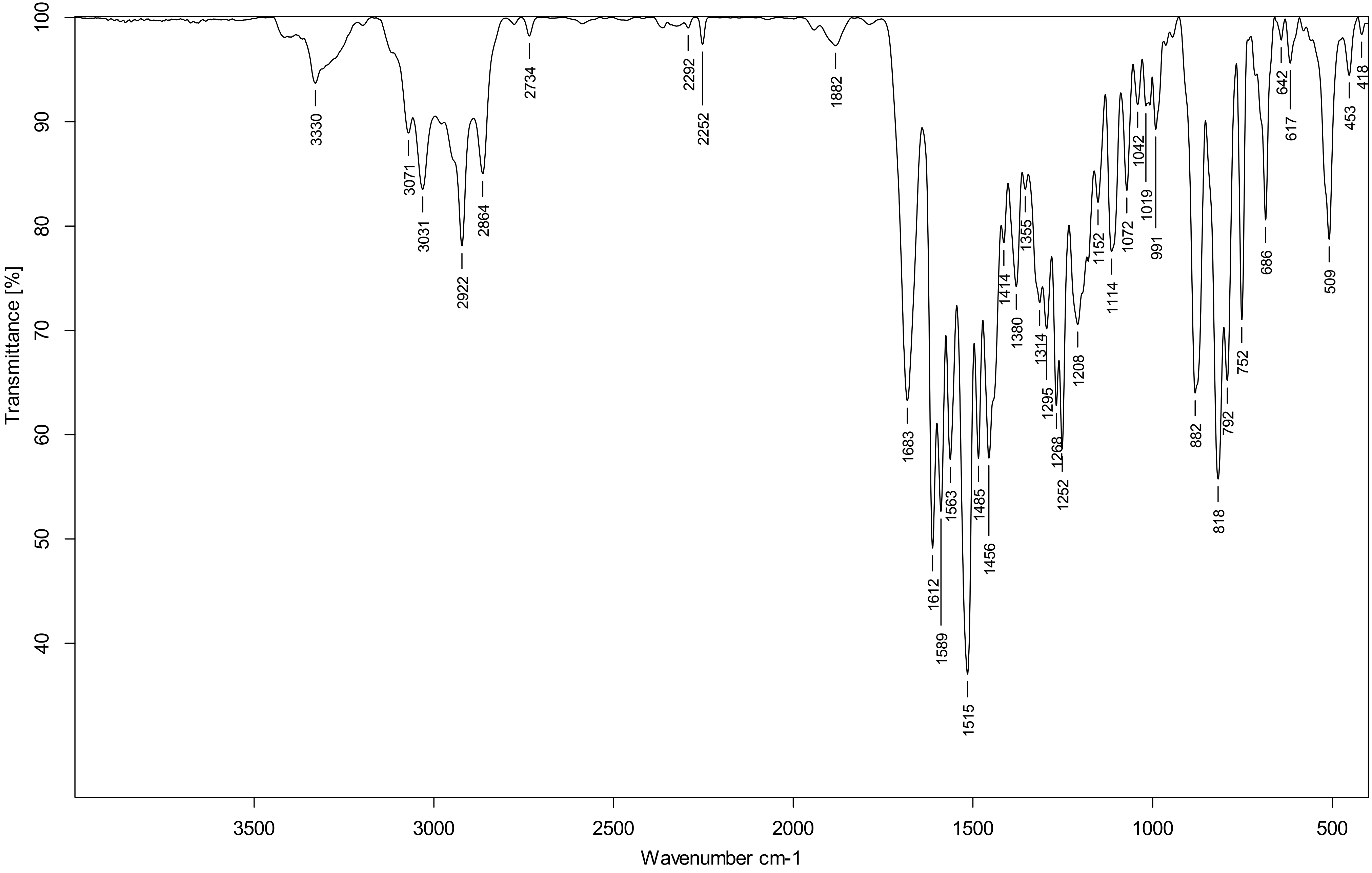

**3f** KBr	18.02.2022

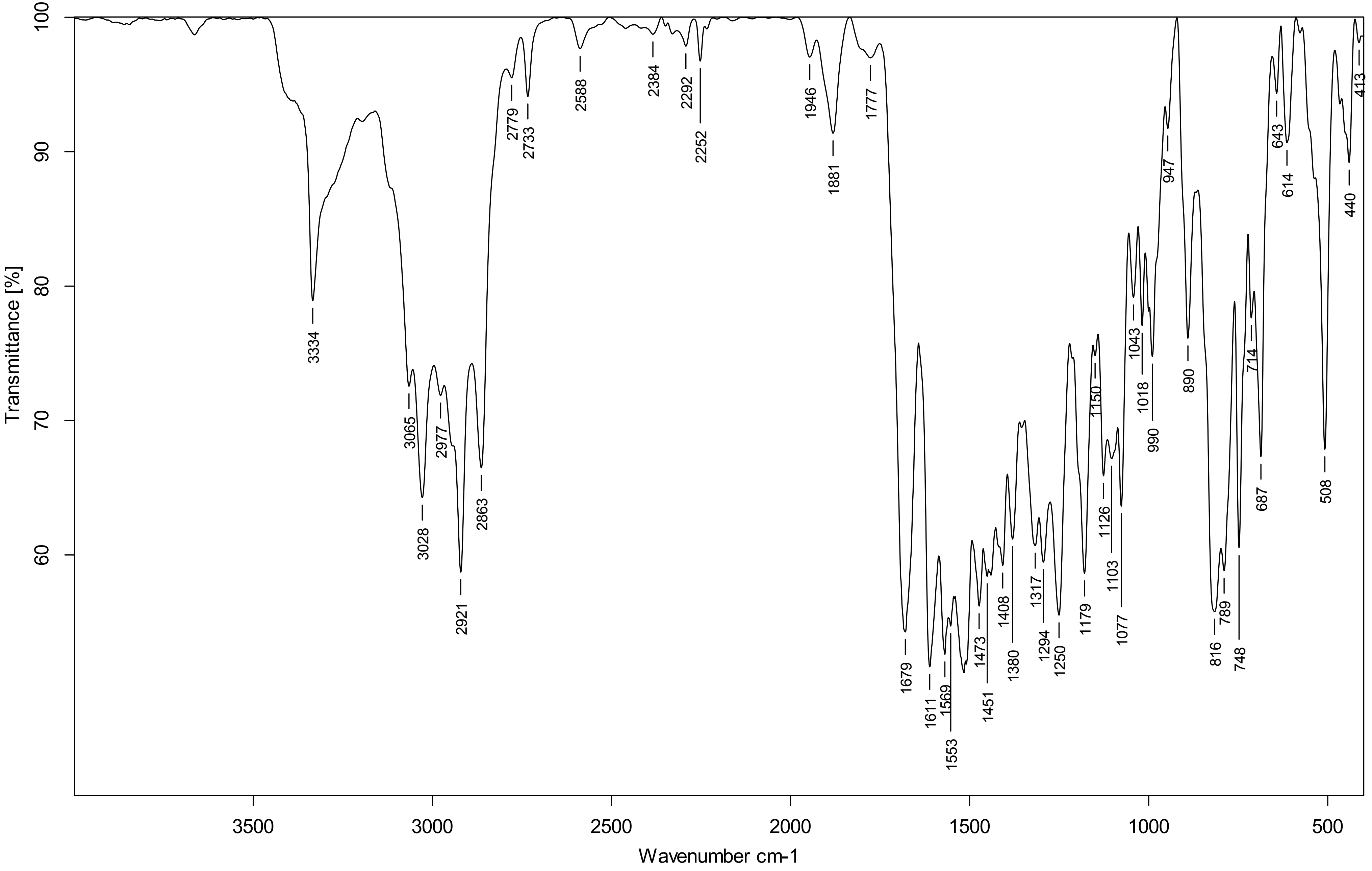

**3g** KBr	18.02.2022

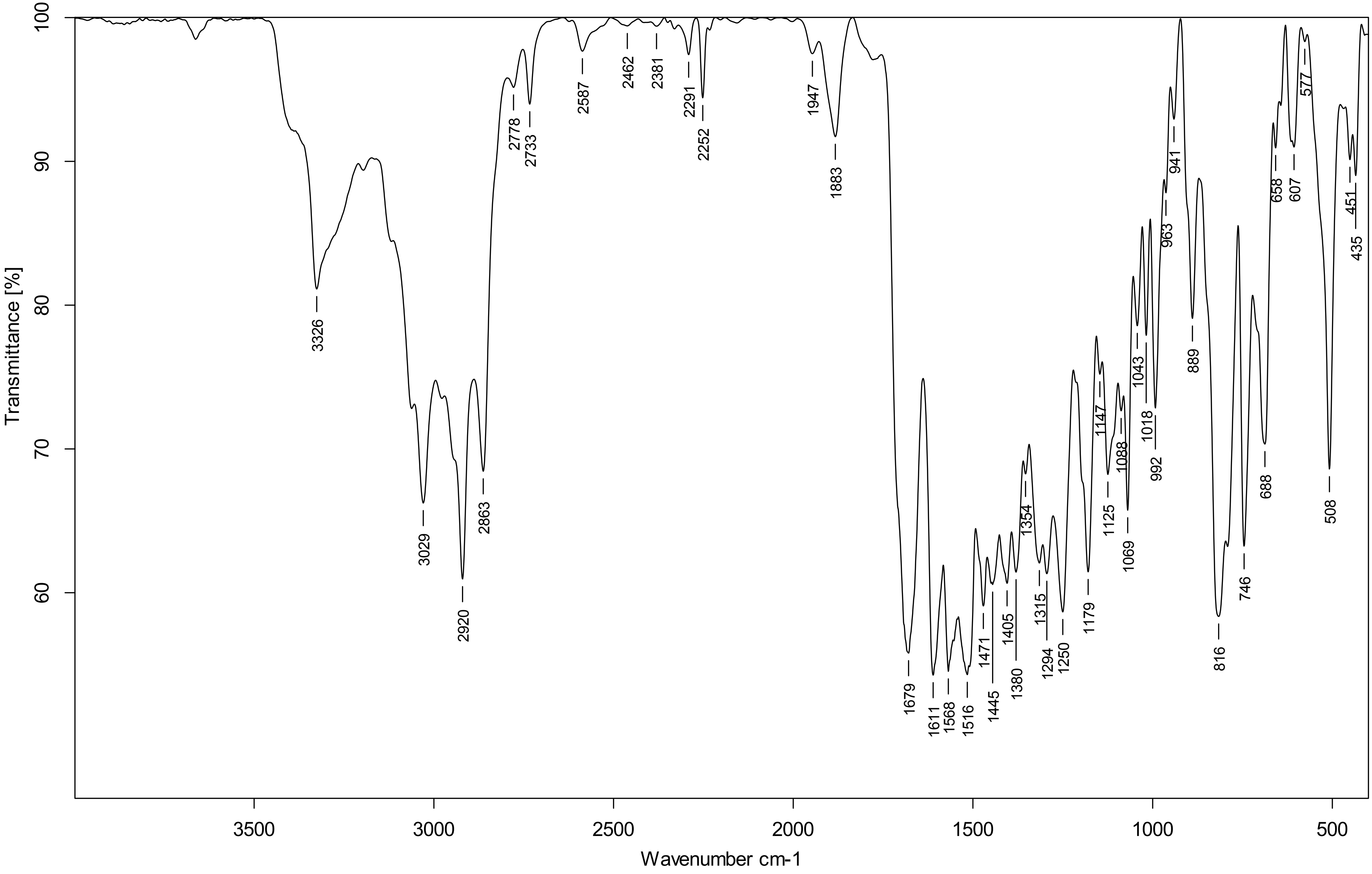

**3h** KBr	18.02.2022

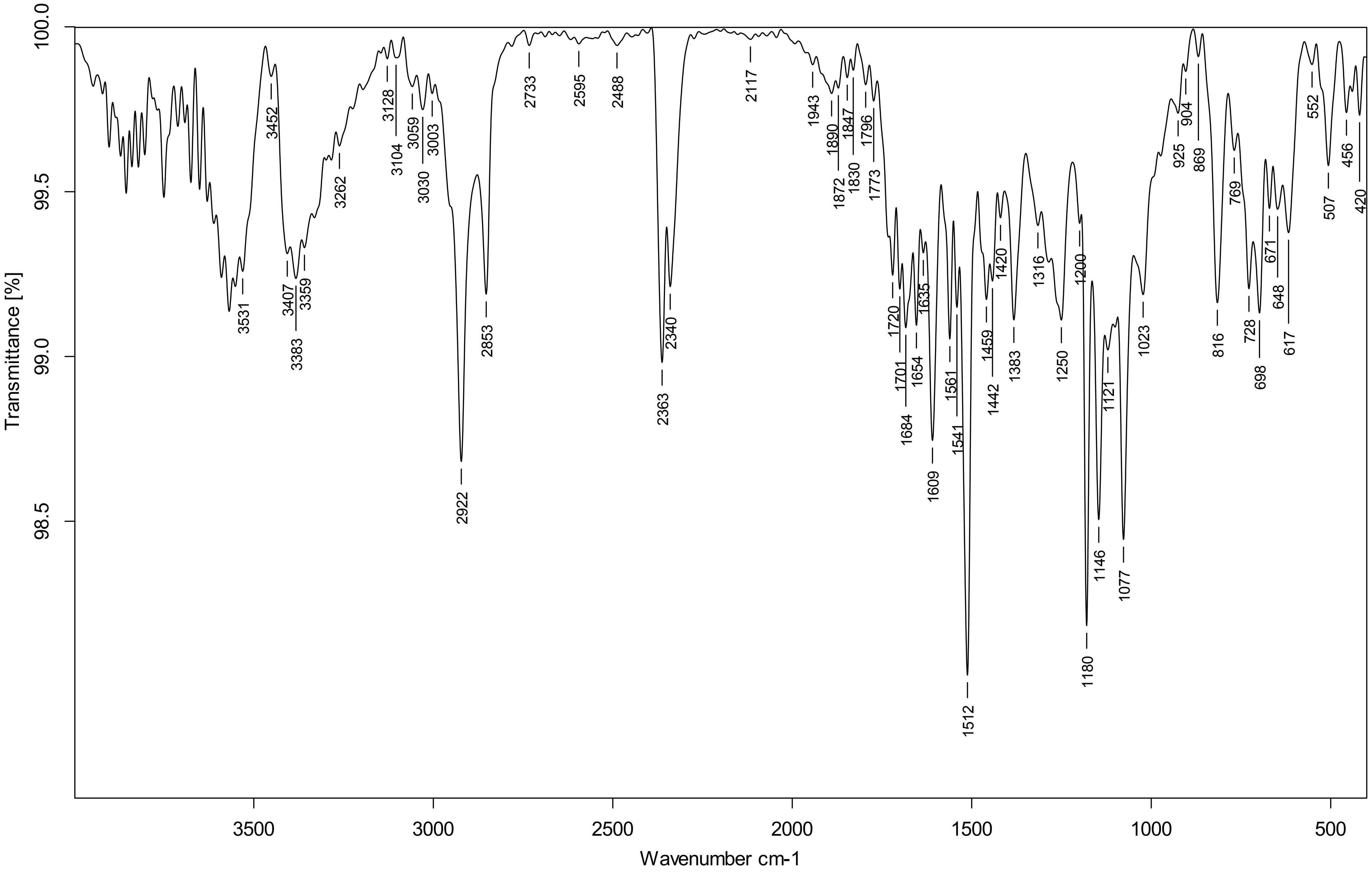

**3i** KBr	18.02.2022

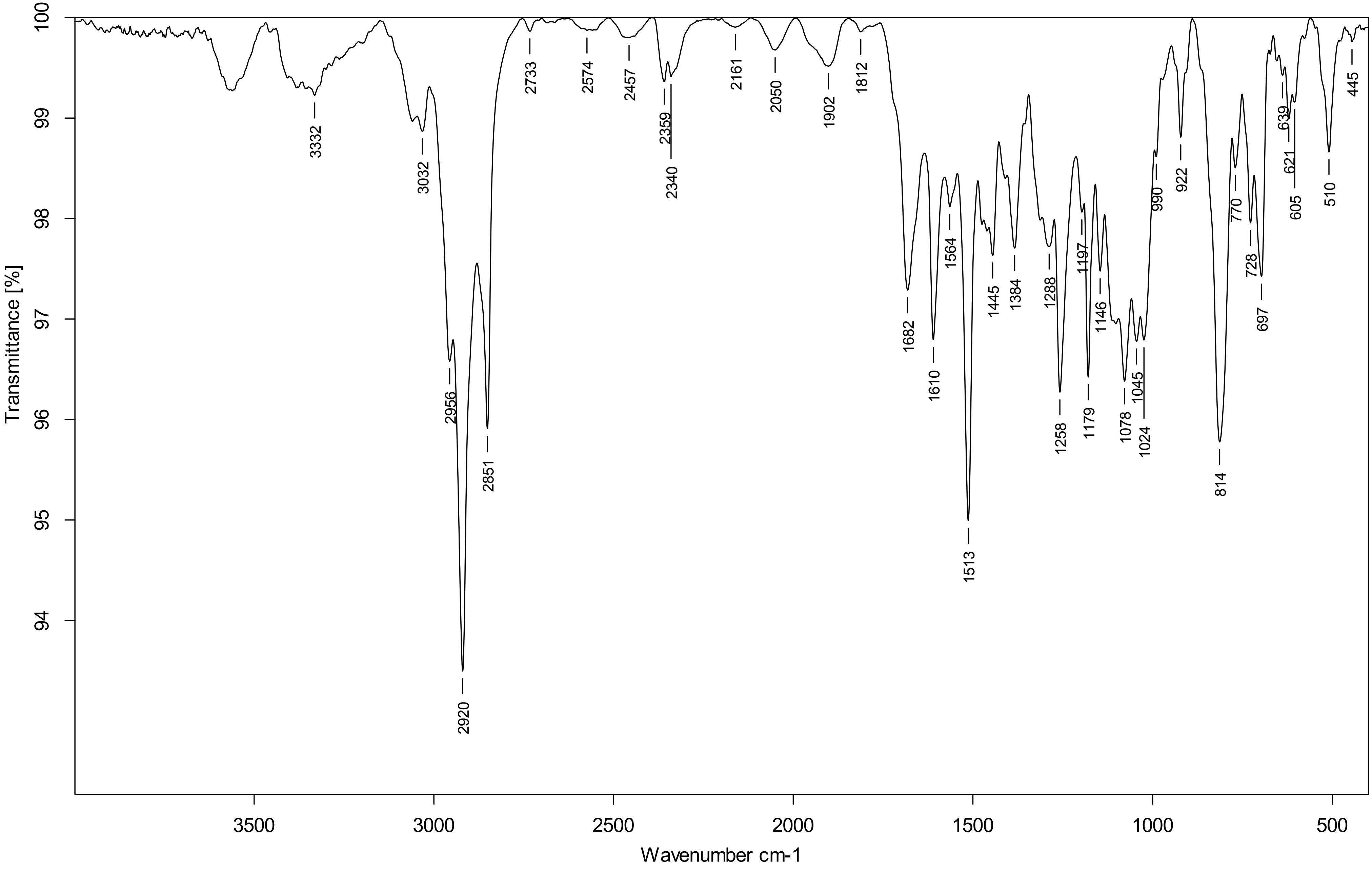

**3j** KBr	18.02.2022

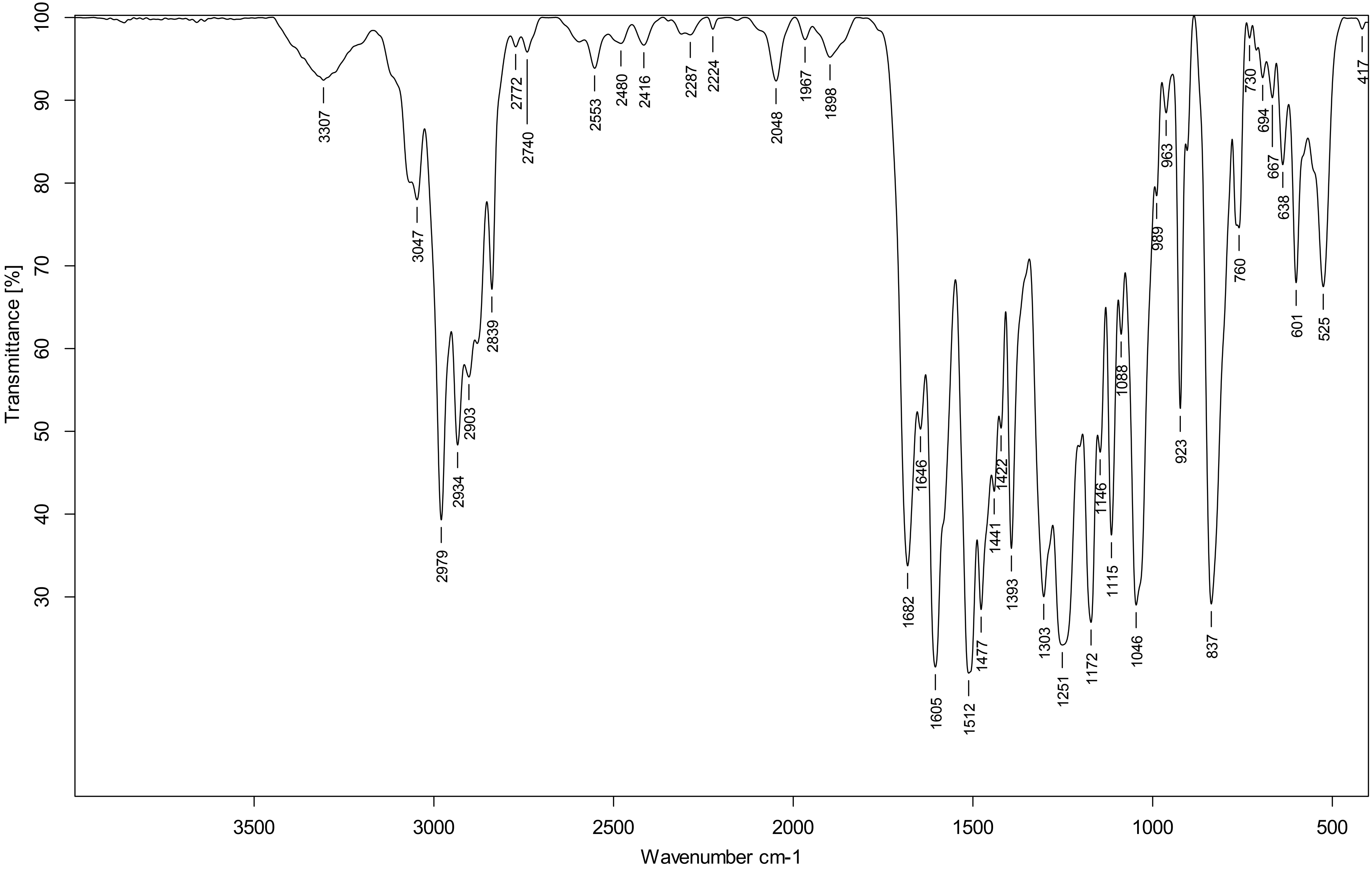

**3k** KBr	18.02.2022
